# Addressing Preconception Behavior Change Through Mobile Phone Apps: Systematic Review and Meta-analysis

**DOI:** 10.2196/41900

**Published:** 2023-04-19

**Authors:** Loretta Musgrave, Kate Cheney, Edwina Dorney, Caroline S E Homer, Adrienne Gordon

**Affiliations:** 1 Centre for Midwifery, Child and Family Health University of Technology Sydney Sydney Australia; 2 Faculty of Medicine and Health The University of Sydney Sydney Australia; 3 Sydney Institute for Women, Children and Families Sydney Local Health District NSW Health Sydney Australia; 4 Burnet Institute Melbourne Australia

**Keywords:** apps, mobile, preconception, prenatal care, perinatal, reproductive health, reproductive age, maternal, interconception, behavior change, mobile phone

## Abstract

**Background:**

Positive health behavior changes before pregnancy can optimize perinatal outcomes for mothers, babies, and future generations. Women are often motivated to positively change their behavior in preparation for pregnancy to enhance their health and well-being. Mobile phone apps may provide an opportunity to deliver public health interventions during the preconception period.

**Objective:**

This review aimed to synthesize the evidence of the effectiveness of mobile phone apps in promoting positive behavior changes in women of reproductive age before they are pregnant (preconception and interconception periods), which may improve future outcomes for mothers and babies.

**Methods:**

Five databases were searched in February 2022 for studies exploring mobile phone apps as a prepregnancy intervention to promote positive behavior change. The identified studies were retrieved and exported to EndNote (Thomson Reuters). Using Covidence (Veritas Health Innovation), a PRISMA (Preferred Reporting Items for Systematic Reviews and Meta-Analyses) study flow diagram was generated to map the number of records identified, included, and excluded. Three independent reviewers assessed the risk of bias and conducted data extraction using the Review Manager software (version 5.4, The Cochrane Collaboration), and the data were then pooled using a random-effects model. The Grades of Recommendation, Assessment, Development, and Evaluation system was used to assess the certainty of the evidence.

**Results:**

Of the 2973 publications identified, 7 (0.24%) were included. The total number of participants across the 7 trials was 3161. Of the 7 studies, 4 (57%) included participants in the interconception period, and 3 (43%) included women in the preconception period. Of the 7 studies, 5 (71%) studies focused on weight reduction, assessing the outcomes of reductions in adiposity and weight. Of the 7 studies, nutrition and dietary outcomes were evaluated in 2 (29%) studies, blood pressure outcomes were compared in 4 (57%) studies, and biochemical and marker outcomes associated with managing disease symptoms were included in 4 (57%) studies. Analysis showed that there were no statistically significant differences in energy intake; weight loss; body fat; and biomarkers such as glycated hemoglobin, total cholesterol, fasting lipid profiles, or blood pressure when compared with standard care.

**Conclusions:**

Owing to the limited number of studies and low certainty of the evidence, no firm conclusions can be drawn on the effects of mobile phone app interventions on promoting positive behavior changes in women of reproductive age before they are pregnant (preconception and interconception periods).

**Trial Registration:**

PROSPERO CRD42017065903; https://tinyurl.com/2p9dwk4a

**International Registered Report Identifier (IRRID):**

RR2-10.1186/s13643-019-0996-6

## Introduction

### Background

Many of the adverse outcomes experienced by mothers and babies in the short term and long term are directly related to the mothers’ health before pregnancy [1-4]. The preconception period is a unique window of opportunity when women are often more motivated to optimize their health and change their lifestyle in preparation for pregnancy [5]. By reducing health and behavioral risks before conception, preconception care prevents pregnancy-related issues from occurring [6]. Modifiable lifestyle behaviors are often assessed through biochemical and anthropometric measurements, self-reporting, and validated tools [7].

Mobile phone apps have the potential to support modifiable behavioral changes known to increase positive health outcomes such as weight loss, physical activity, and balanced diet [8]. Smartphones are mobile phones that operate many computer functions, usually having a high-resolution touchscreen interface, internet access, Wi-Fi connectivity, web-browsing capabilities, and an operating system that can run and download apps [9]. Globally, in the first quarter of 2020, health and fitness mobile and internet applications were downloaded 593 million times [10]. The COVID-19 pandemic has led to an increase in internet-based care and self-care via telehealth and mobile health in all areas of health, including reproductive and women’s health [11].

A recent randomized controlled trial (RCT) by Sandborg and Söderström [12] provided an example of how a smartphone app intervention (HealthyMoms) could be used to promote healthy weight gain, healthy diet, and physical activity during pregnancy. Although the authors did not find a statistically significant effect on gestation weight gain, they did see that women who were overweight or obese before pregnancy in the intervention group gained fewer kilograms than those in the control group in the imputed analyses (−1.33 kg, 95% CI −2.92 to 0.26; *P*=.10) and the completers-only analyses (−1.67 kg, 95% CI −3.26 to −0.09; *P*=.03) [12].

Another example that demonstrates how an app may be used in pregnancy to change behavior is a study by Kennelly et al [13,14]; the primary outcome of this RCT was to evaluate the effect of a prenatal app on the incidence of gestational diabetes mellitus (GDM) in overweight and obese women. Although the app did not decrease the incidence of GDM, a follow-up study of the secondary outcomes [15] of nutrition, behavior change, and physical activity showed that apps could be a prenatal intervention for improving maternal health behaviors [15]. Physical activity (metabolic equivalent of a task—min/wk) was higher in the app group after intervention (mean difference [MD] 141.4, 95% CI 62.9-219.9), and the proportion of women at the *maintenance* stage of change for physical activity was higher in the intervention group (56.3% vs 31.2%) [15].

Poor diet before, during, or after pregnancy can lead to compromised fetal and infant growth and poorer birth outcomes in babies [16]. A healthy diet before conception has been associated with a lower risk of gestational diabetes, hypertension, pre-eclampsia, and preterm delivery [16]. A systematic review by Overdijkink et al [17] found that mobile health apps for supporting lifestyle and pregnancy care in high-income countries can reduce gestational weight gain, increase the intake of vegetables and fruit, and aid in smoking cessation [17]; however, evidence is lacking on the effect of such apps during the preconception or interconception period.

Although women are often keen to optimize their health before conception, many women do not plan pregnancy and, therefore, miss the opportunity to make positive changes to their health; therefore, taking a life-course perspective may be advantageous, and using smartphone apps to deliver interventions is a potential strategy that could be implemented to reach many people very quickly [18].

### Objectives

The primary objective of this study was to review the evidence of the effectiveness of mobile phone apps in supporting positive behavior changes in women of reproductive age before they are pregnant (preconception and interconception periods), which may improve future outcomes for mothers and babies. Our secondary objectives were to determine the effects of mobile apps on self-efficacy and psychosocial and general health outcomes.

## Methods

A systematic review was conducted according to the PRISMA (Preferred Reporting Items for Systematic Reviews and Meta-Analyses) statement [19]. The protocol was registered with the International Prospective Register of Systematic Review (PROSPERO; CRD42017065903) and published in 2019 [20].

### Criteria for Considering Studies for This Review

#### Types of Studies

RCTs, quasi-RCTs, and cluster-randomized trials that aimed to assess the effects of mobile app–based interventions on the knowledge or behavior of women of reproductive age were considered for inclusion.

#### Types of Participants

Our study population included nonpregnant women of reproductive age, regardless of whether they were planning a pregnancy. The term “preconception” is a broad concept that is understood differently by different individuals and couples who are in the prepregnancy period. In defining preconception, we included the provision of pre-emptive, promotive, or therapeutic health before conception or between 2 pregnancies (interconception) [21]. We have used the “potential preconception perspective” definition by Hill et al [22]; 4 defining elements characterize this perspective: (1) reproductive age: (2) a man or woman; (3) a woman or partner who is not pregnant; and (4) only sexually active individuals, including those who partake in intercourse without using effective contraception and those who experience contraceptive failure [22]. To determine the inclusion and exclusion criteria, we used the United Nations Department of Economic and Social Affairs (2020) family planning delineation of reproductive age—15 to 49 years [23].

#### Types of Interventions

Mobile app interventions were included if they provided general information for women of reproductive age or focused on a specific risk factor relevant to future perinatal outcomes. Interventions that supported information delivery, decision-making, self-care, and behavior change or risk reduction strategies or advice were included. There were no restrictions regarding who developed or funded the intervention.

Trials that assessed behavior change interventions, self-management of wellness, and disease prevention management (single or combined) were included. Studies published as abstracts only were included if sufficient information was available or if we obtained the required information by contacting the authors.

Individualized interventions with capabilities such as self-monitoring, intention formation, specific goal setting, and review of and feedback on goals were included. Studies were excluded for several reasons, which are published in the protocol [20].

#### Comparisons

Our review aimed to assess the following comparisons: (1) mobile phone apps versus SMS text messaging–based or paper-based communications, for example, comparing an app that could be tailored to the individual versus a text-based intervention that provided general information; (2) mobile phone apps versus face-to-face or telephone conversations, for example, an app that did not have any health care professional (HCP) interaction versus a personal interaction with an HCP; and (3) mobile phone apps versus usual or standard care as described by the authors or no specific intervention, for example, an app designed as the intervention for the study versus the provision of care from an HCP or no provision of care.

#### Outcomes

We sought studies that evaluated targeted interventions, such as pregnancy planning support and advice about healthy weight, diet, exercise, reduction or cessation of smoking, and alcohol and drug use. We also looked for studies that had interventions for supporting decision-making or for addressing specific physical and psychosocial needs such as perinatal mental health, for example, anxiety and depression. Furthermore, we searched for studies that evaluated health service use and outcomes specific to unintended and intended pregnancies (both maternal and neonatal; Table 1).

**Table 1:** Primary and secondary outcomes.

	Behavior changes as defined by the trial authors relative to the goal of the intervention
Primary outcomes	Healthier lifestyle choices Reduced at-risk behaviors, for example, smoking cessation and alcohol intake cessation or reduction Increase in physical activity Weight control or reduction in adiposity Diabetes management, that is, blood glucose control Improved nutrition Optimum management of disease symptoms, for example, reduction in blood pressure in hypertensive disease or management of thyroid disease Reduction in unwanted pregnancies
Secondary outcomes	Self-efficacy (as defined by the trial authors using a validated scale such as the Rosenberg Self-Esteem Scale) Psychosocial outcomes such as depression and anxiety (as defined by the trial authors and measured using a validated tool, eg, Cambridge Worry Scale, State-Trait Anxiety Index, or Edinburgh Depression Scale) General health (as defined by the trial authors using a standardized measure such as a general health assessment tool) Knowledge of the targeted intervention topic, for example, the biomedical, social, or environmental risk (as defined by the trial author) Evaluation of the intervention (as reported by the trial authors, eg, adherence to lifestyle recommendations) Health service use (as reported by the trial authors, eg, outpatient clinic appointment for the management of health or lifestyle, interaction with health service programs, interaction with GP^a^ services, and the use of inpatient services or length of stay in hospital)
Outcomes specific to unintended pregnancy	Pregnancy intention (mistimed, ambivalent, or as reported by the trial authors, eg, a psychometrically valid measure of pregnancy intention that assesses intention on a continuous scale, such as the London Measure of Unplanned Pregnancy) Health service use (as reported to the trial authors, eg, family planning clinic, contraceptive counseling, pregnancy test referral, and abortion options or services)
Outcomes specific to pregnancy—maternal	Maternal morbidity (major)—a combination of near-miss mortality and unexpected admission to the intensive care unit or death, as defined by the WHO^b^ Antepartum hemorrhage Postpartum hemorrhage Gestational diabetes Pre-eclampsia Mode of birth Induction of labor Pain relief in labor Successful initiation of breastfeeding Maternal satisfaction Antenatal or postnatal depression Early pregnancy loss (miscarriage) Unanticipated admission to the hospital postnatally
Outcomes specific to pregnancy—neonatal	Perinatal morbidity (major—unexpected admission to intensive care unit) Stillbirth Neonatal death Mode of birth Gestational age at birth SGA^c^—birth weight <the 10th percentile (using the growth chart defined by the trialist) LGA^d^—birth weight >the 90th percentile (using the growth chart defined by the trialist) Infant feeding method at 3 months Unanticipated admission to the hospital

^a^GP: general practitioner.

^b^WHO: World Health Organization.

^c^SGA: small for gestational age.

^d^LGA: large for gestational age.

#### Electronic Searches

The initial search was conducted on February 4, 2021, using index terms. The search was repeated before the final analysis, on February 14, 2022, and no further studies were retrieved. To avoid missing nonindexed concepts, electronic searches using subject headings and all fields for keywords were conducted (Multimedia Appendix 1). Systematic searches were performed using 5 electronic bibliographic databases: Cochrane Central Register of Controlled Trials (CENTRAL), MEDLINE (Ovid), Embase, CINAHL (EBSCO), and Web of Science. In addition, we searched ClinicalTrials.gov and the World Health Organization International Clinical Trials Registry Platform (Global Index Medicus) for unpublished, planned, and ongoing trial reports. No language or date restrictions were applied. Abstracts and full-length articles were obtained for each citation, where available.

#### Searching Other Resources

We hand-searched the reference lists of the included ongoing studies and relevant reviews identified through electronic searches to identify unpublished trials. We then emailed the trial contact for ongoing or completed but unpublished trials for further information.

### Data Collection and Analysis

#### Study Selection

**Figure 1:**
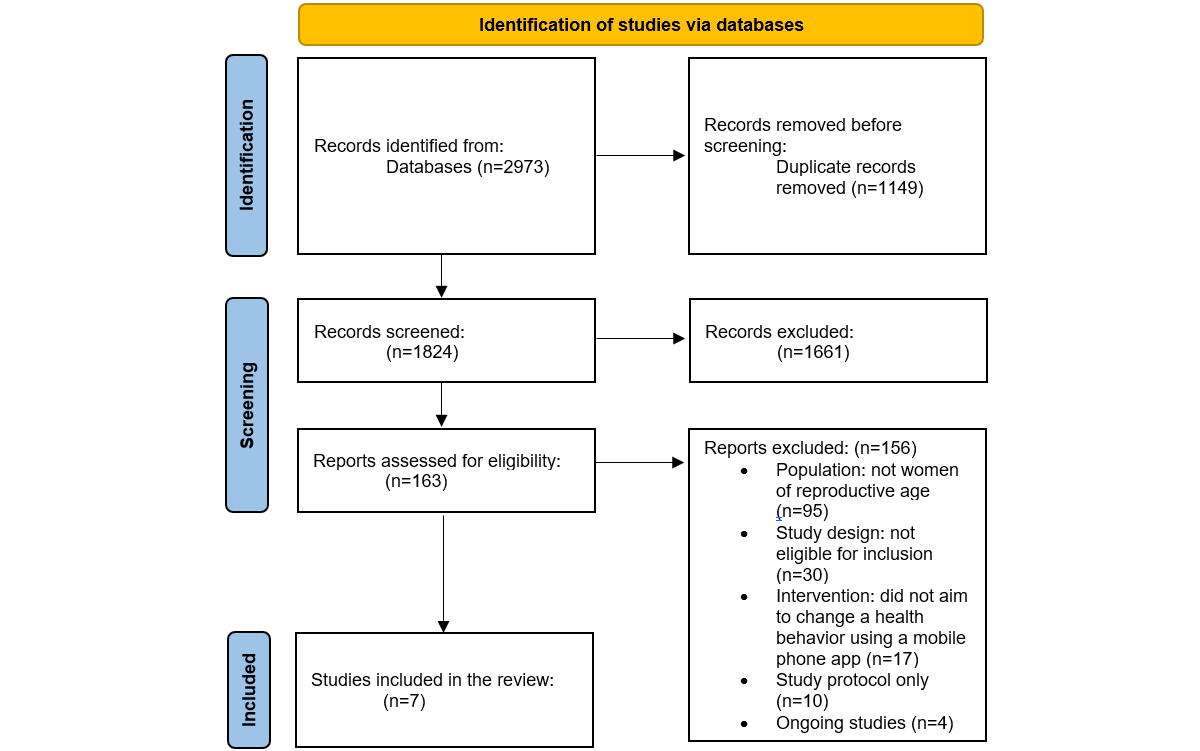
PRISMA (Preferred Reporting Items for Systematic Reviews and Meta-Analyses) 2020 flow diagram for new systematic reviews, which includes searches of databases [19].

All the identified studies were retrieved from web-based databases and exported to the reference management system EndNote (version X8; Thomson Reuters). All remaining citations and abstracts were uploaded to the Covidence systematic review software (Veritas Health Innovation). A PRISMA [19] study flow diagram was generated in Covidence to map out the number of records identified, included, and excluded (Figure 1).

#### Data Extraction and Management

Data related to study identification, methods, population, interventions, comparisons, and outcomes were extracted using the Covidence systematic review software, and the authors were not blinded to journal titles or study authors or institutions throughout the process. Studies were screened by LMM, KC, ED, and AG based on the titles and abstracts. After screening, the full texts were retrieved and reviewed by LMM and 2 other authors (KC and ED). Disagreements were resolved through discussion with a fourth investigator (AG).

#### Assessment of Risk of Bias in the Included Studies

The Cochrane risk of bias tool was applied to the included studies [24] using the following domains: (1) sequence generation, (2) allocation concealment, (3) blinding of participants and personnel for all outcomes, (4) blinding of outcome assessors for all outcomes, (5) incomplete outcome data for all outcomes, (6) selective outcome reporting, and (7) other sources of bias. Two reviewers independently assessed the studies (KC and ED) to reach a consensus, and a third reviewer resolved disagreements (LMM). Studies were rated as having “high,” “low,” or “unclear” risk of bias.

#### Measurement of Treatment Effect

The Review Manager software (version 5.4; The Cochrane Collaboration) was used for statistical analyses [25]. The individual differences and MD were the units of analysis. Relative risks and risk differences were used to measure the effectiveness of the intervention between the groups.

#### Assessment of Heterogeneity

Heterogeneity was considered by assessing the participants (women of reproductive age), intervention (mobile phone app), and primary outcome (behavior as defined by the trial authors) to determine whether they were sufficiently similar to be combined. Statistical heterogeneity was evaluated through the visual inspection of the CI of amalgamated studies for overlap, and the *I*^2^ statistic [24] and funnel plots were generated; however, because of the limited number of studies incorporated, no publication bias was detected.

#### Data Synthesis

We used the Review Manager software (version 5.4) [25] to conduct a meta-analysis of the results from the included studies; this was achieved using a fixed-effects model. Continuous data were determined using the MD. A meta-analysis was performed on 8 outcomes.

#### Subgroup Analysis

Although a subgroup analysis was planned, there were insufficient data to conduct this exploration.

### Sensitivity Analyses: Quality of Evidence

A sensitivity analysis was not conducted because of the small number of trials for each outcome.

The Grades of Recommendation, Assessment, Development, and Evaluation approach was used to evaluate the quality of the body of evidence [26]. Study limitations, consistency of effect, imprecision, indirectness, and publication bias were considered for specific outcomes, and the evidence was graded accordingly [26]. The web-based GRADEpro Guideline Development Tool [23] was used to construct a summary of findings table.

## Results

### Description of the Search

#### Overview

The search strategy used for this review is described using PRISMA and presented in Figure 1 [19]. One of the authors (LMM) searched the databases on February 14, 2022; the search returned 2973 references, of which 1149 (38.65%) duplicates were removed. The titles and abstracts of 61.35% (1824/2973) of the studies were screened by 2 authors independently (among LMM, KC, and ED), and 91.06% (1661/1824) of these studies were excluded after an assessment based on study type, population, and relevance. Two authors independently reviewed 163 full texts against the inclusion and exclusion criteria; at this time, hand-searching was performed by LMM; however, no additional studies meeting the inclusion criteria were discovered. Excluded studies (n=156) are presented in Multimedia Appendix 2; several studies had multiple reasons for exclusion; however, each was allocated to a primary category. In total, 7 trials met the inclusion criteria (Multimedia Appendix 3 [27-33]), and 4 trials were registered as ongoing (Multimedia Appendix 4). Disagreements between the authors were resolved through consultation with a third reviewer (AG) throughout the process.

#### Excluded Studies

A table of the excluded studies and reasons for exclusion are provided in Multimedia Appendix 2. Studies excluded as “ongoing studies” (n=4) are presented in Multimedia Appendix 4.

#### Included Studies

The 7 included studies are summarized in Table 2. The details of the characteristics of these trials are provided in Multimedia Appendix 3. These studies were published from 2017 to 2022.

All the 7 studies used a parallel-group design, and 1 study (14%) used a single center. All studies recruited participants from a health care setting (hospital or health care clinic or center). All the 7 studies compared women using a smartphone app with routine care from high-income countries, except for 1 study (14%) from Iran, which is considered a lower-middle–income country. All studies compared a mobile app with standard (routine) care. One trial used a mobile phone app and face-to-face coaching versus standard care [27], whereas another trial combined several intervention components and compared these with the standard care group [28].

The studies assessed a wide variety of outcome variables that were considered measures of behavior change. These included a change in weight control, a reduction in adiposity, a reduction in blood pressure, a reduction in glycated hemoglobin (HbA_1c_), a reduction in fasting lipid profiles, smoking or alcohol reduction or cessation, an increase in physical activity, a reduction in sedentary time, and an improvement in nutrition compared with standard care or no specific intervention. Of the 7 studies, 5 (71%) studies included ≥1 anthropometrics (BMI, body fat percentage, weight loss, waist circumference [WC], and hip circumference). Of the 7 studies, blood pressure was measured in 4 (57%) studies, and a range of biochemical tests and markers were associated with managing disease symptoms, such as the oral glucose tolerance test, HbA_1c_, lipid profiles, liver function, and total cholesterol, high-density lipoprotein (HDL), and triglycerides. Of the 7 studies, nutrition and dietary outcomes were assessed in 2 (29%) studies. Kilojoules, caloric, and macronutrient intake data were collected for these variables and then outcomes were compared for both the control and intervention arms of the studies. Only 2 studies of the 7 (29%) used the dietary risk scores (DRSs) to determine a change in dietary outcomes.

**Table 2:** Summary of the included studies (N=7).

Study	Mobile phone app	Location	Randomized participants, n	Type of participants randomized	Intervention	Control	Primary outcome
Gilmore et al [29], 2017	E-Moms	United States	40	Women in their postpartum (interconception) period who were overweight or obese	E-Moms app (n=19)	Standard care (WIC^a^ Moms; n=16)	Postpartum weight loss (change in weight following the 4-month intervention)
Bijlholt et al [27], 2021	INTER-ACT^b^	Belgium	1450	Women with excessive gestational weight gain in the period preceding pregnancy (interconception)	INTER-ACT app and face-to-face coaching (n=724)	Standard care (n=726)	Eating behavior, energy intake (kcal), physical activity (MET^c^—min/wk), and sedentary time (min/d) measured at 6 and 12 months post partum
Jannati et al [30], 2020	Happy Mom	Iran	78	Women in their postnatal (interconception) period	Happy Mom app (n=38)	Standard care (n=37)	Change in EPDS^d^ from baseline
Lim et al [31], 2021	SPAROW^e^ Trial	Singapore	200	Postnatal women who had been diagnosed with gestational diabetes mellitus (interconception)	*nBuddy* app (n=101)	Standard care (n=99)	Weight loss—return to first trimester weight measured at 4 months post partum
Oostingh et al [32], 2020	Smarter Pregnancy	Netherlands	848	Women attending an IVF^f^ clinic (preconception)	Smarter Pregnancy app version with personalized interaction and emails (n=414)	Smarter Pregnancy app “light” version (not tailored; n=434)	Improvement in DRS^g^ at 24 weeks after starting program
van Dijk et al [33], 2020	Smarter Pregnancy	Netherlands	218	Women who are contemplating pregnancy or already pregnant (<13 weeks of pregnancy) and attending urban health services (preconception)	Smarter Pregnancy app version with personalized interaction (n=109)	Smarter Pregnancy app version with limited functionality and no personalized interaction (n=109)	Improvement in DRS at 24 weeks after starting the program
Hanafiah et al [28], 2022	Jom mama^h^	Malaysia	549	Newly registered married or engaged women (young women before their first pregnancy; preconception)	Interaction with community health promotors (HCP^i^), an app, and a web-based interface (n=272)	Standard care (n=276)	Efficacy of a complex behavioral change intervention in enhancing women’s health before pregnancy

^a^WIC: women, infants, and children.

^b^INTER-ACT: A randomized controlled trial that uses a lifestyle intervention that combines a mobile phone app and face-to-face coaching sessions between 6 weeks and 6 months postpartum.

^c^MET: metabolic equivalent of task.

^d^EPDS: Edinburgh Postnatal Depression Scale.

^e^SPAROW: Smartphone App to Restore Optimum Weight randomized controlled trial.

^f^IVF: in vitro fertilization.

^g^DRS: dietary risk score.

^h^Jom mama: a randomized controlled trial that used a complex preconception intervention that included a mobile phone app.

^i^HCP: health care professional.

#### Risk of Bias Assessment of the Included Studies

The risk of bias assessment for each included study is presented in Multimedia Appendix 3. Table 3 summarizes the risk of bias for each study individually. To assess the risk of bias due to selective outcome reporting, trial registrations and protocols were checked to validate that the intended outcomes were reported. The reporting bias across all studies was low, with all reporting data for the primary outcomes.

**Table 3:** The risk of bias summary: review authors’ judgments about each risk of bias item of each included study.

Study (publication year)	Risk assessment						
	Random sequence generation	Allocation concealment	Blinding of participants and personnel	Blinding of outcome assessors	Incomplete outcome data	Selective reporting	Other bias
Bijlholt et al [27], 2021	Low	High	High	Unclear	Low	Low	High
Gilmore et al [29], 2017	High	High	High	High	Low	Low	Unclear
Jannati et al [30], 2020	Low	High	High	High	Unclear	Low	High
Lim et al [31], 2021	Low	Low	High	High	Low	Low	Unclear
Oostingh et al [32], 2020	Low	Low	Low	Low	Low	Low	Unclear
Hanafiah et al [28], 2022	Low	Low	High	Unclear	Low	Low	Unclear
van Dijk et al [33], 2020	Low	Low	High	High	Unclear	Low	Unclear

### Description of Participants

The total number of participants across the 7 trials was 3161. Of the 7 trials, 3 (43%) recruited women planning pregnancy (preconception; n=1393), and 4 (57%) recruited women in their postpartum (interconception) period (n=1768). Of the 7 trials, 2 (29%) trials included women in their postpartum (interconception) period who were considered overweight or obese (n=1490). Of the 7 trials, 1 (14%) trial included women <13 weeks pregnant (n=73); we removed this subgroup of women before our analysis, and participant characteristics are presented in Multimedia Appendix 3.

### Description of Interventions

All mobile phone apps were purposely designed for individual studies and free to participants. No data were presented regarding intervention modifications in any of the included studies. Data regarding the cost-benefit analysis of the interventions were not apparent in any of the studies.

### Effects of Interventions

A “summary of findings” table for the main comparison, that is, between the mobile phone app and standard care, can be found in Multimedia Appendix 5.

### Primary Outcomes

#### Overview

The primary outcome of interest was a change in behaviors as defined by the trial authors comparative to the aim of the intervention (Table 1). The results are presented using the measures used by the authors to assess behavior change; for example, weight control or reduction in adiposity was measured by assessing energy intake (kcal), BMI, and weight loss (kg). A variety of biochemical measures were also used by the authors to assess changes; for example, weight control was measured using fasting lipids, and the management of disease symptoms was measured by assessing HbA_1c_ and liver function test variables.

#### Weight Control or Reduction in Adiposity

##### Weight Control—Reduction in Calories and Improved Diet

There was no significant MD in reduction in calories (kcal) between women who received the mobile phone app and those who did not (MD −140.89 less, 95% CI −190.19 to 91.59; 2 trials, 937 women; *I^2^*=98%; very low certainty evidence). Bijlholt et al [27] also found that sugar was lower in the total caloric intake in the app group (adjusted MD −0.019, 95% CI −0.028 to −0.010; *P*<.001), and this was significant (refer to Figure S1 in Multimedia Appendix 6 [27,31]).

A study by Lim et al [31] compared an intervention that aimed to assist women in returning to their ideal weight post partum using a mobile phone app with standard care. At 4 months, the intervention group reported reductions in total caloric intake (−614.2 kcal, 95% CI −751.5 to −476.9), total fat (−20.7 g, 95% CI −27.2 to −14.2), and sugar (−27.9 g, 95% CI −35.7 to −20.1) when compared with the control group.

Bijlholt et al [27] compared a smartphone app with standard care in women with excessive gestational weight gain in the preceding pregnancy. At 6 months post partum, “restrained eating” score was 1 point higher in the intervention group (95% CI 0.5-1.5; *P*<.001), and “uncontrolled eating” was 1 point lower in the control group (95% CI −1.9 to −0.2; *P*=.02). At follow-up, the differences were no longer statistically significant.

Two studies assessed changes in eating behavior using DRS. The primary outcome of the study by Oostingh et al [32] was an improvement in good nutritional behaviors based on a reduction in DRS 24 weeks after starting the program and 12 weeks after the completion of the program in women undergoing in vitro fertilization treatment. DRS is calculated as the sum of scores for vegetable, fruit, and folic acid supplement intake (range 0-9); the higher the score, the more adequate the nutritional intake and behaviors are. DRSs at 24 weeks (β=.779, 95% CI 0.456-1.090) and 36 weeks (β=.816, 95% CI 0.478-1.142) showed no significant difference. In the study by van Dijk et al [33], participants in the intervention group documented a nonsignificant reduction in DRS (β=.750, 95% CI 0.188-1.341) compared with the control group at 24 weeks [33].

##### Weight Control—Reduction in Weight

There was no significant MD in weight loss (kg) between women who received the mobile phone app and those who did not (MD −0.78 less, 95% CI −1.20 to −0.36; 3 trials, 529 women; *I^2^*=94%; very low certainty evidence; Figure S2 in Multimedia Appendix 6 [28,29,31]).

##### Weight Control—Reduction in WC and Waist to Hip Ratio

One study compared the change in WC and another study measured the change in waist to hip ratio; therefore, a meta-analysis could not be performed. The *Jom Mama* study [28] (n=305) measured the change in WC (baseline minus end point assessment) and found that the intervention group had a mean increase in WC by 1.2 (SD 6.6) cm, and the control group had a mean increase in WC by 1.0 (SD 5.6) cm; this difference was not statistically significant. In the study by Gilmore et al [29] (n=35), there was no significant MD in the waist to hip ratio (cm) between women who received the intervention and those who did not (MD −0.01 less, 95% CI −0.02 to 0.00), and the certainty of evidence was considered low [28,29].

##### Reduction in Adiposity—BMI

There was no significant MD in percent body fat BMI between women who received the mobile phone app and those who did not (MD −0.32 less, 95% CI −0.55 to −0.09; 2 trials, 340 women; *I^2^*=98%; low certainty evidence; Figure S3 in Multimedia Appendix 6 [28,29]).

#### Optimizing Health and Improving Chronic Health Disease

##### Reduction in Blood Pressure

There was no significant MD in systolic or diastolic blood pressure (mm Hg) between women who received the mobile phone app and those who did not (systolic: MD −1.63 less, 95% CI −0.42 to 3.68; 3 trials, 529 women; *I^2^*=0%; low certainty evidence; Figure S4 in Multimedia Appendix 6 [28,29,31]; diastolic: MD −1.33 less, 95% CI −0.77 to 3.42; 2 trials, 340 women; *I^2^*=0%; low certainty evidence; Figure S5 in Multimedia Appendix 6 [28,29]).

##### Reduction in Glucose Tolerance, HbA_1c_, and Fasting Lipid Profiles

There was no significant MD in HbA_1c_ (mmol/L) between women who received the mobile phone app intervention and those who did not (MD 0.10, 95% CI 0.04 to −0.16; 2 trials, 494 women; *I^2^*=0%; low certainty evidence; Figure S6 in Multimedia Appendix 6 [28,31]). Lim et al [31] measured glucose tolerance in women diagnosed with gestational diabetes antenatally. The intervention group received an app, whereas the control group received standard care. At 4 months postnatally, 3% of participants in the intervention group and 0% of participants in the control group had impaired fasting glucose, and 14% of participants in the intervention group and 19% of participants in the control group had impaired glucose tolerance [31]. There was no significant MD in total cholesterol (mmol/L; MD 0.02, 95% CI −0.13 to 0.18; 2 trials, 494 women; *I^2^*=0%; low certainty evidence; Figure S7 in Multimedia Appendix 6 [28,31]). There was no significant MD in HDL (mmol/L; MD 0.01, 95% CI −0.06 to 0.08; 2 trials, 494 women; *I^2^*=0%; low certainty evidence; Figure S8 in Multimedia Appendix 6 [28,31]). The *Jom Mama* trial [28] found no difference in the mean triglyceride level (intervention: mean 0.90, SD 0.5; control: mean 0.85, SD 0.4; *P*=.36).

#### Changes in At-Risk Behaviors—Alcohol and Smoking

Only 1 study [32] (14%) reported on alcohol and smoking outcomes (848 women); therefore, a meta-analysis was not conducted, and the certainty of evidence was considered low. Oostingh et al [32] calculated a lifestyle risk score (LRS) and the smoking score and alcohol consumption score that contributed to the LRS. A linear regression model (difference-in-differences) was used to analyze differences in improvements in LRS between the groups and adjusted for baseline values. At the 24-week time point, there was a decrease in LRS in the intervention group (β=.108, 95% CI 0.021-0.203), and at the 36-week end point, LRS was still lower than the baseline scores (β=.067, 95% CI −0.032 to 0.165) [32]. Although there appears to be some effect of the intervention at 24 weeks for both smoking and alcohol consumption, this effect appears to be washed out at 36 weeks after the program [32].

#### Changes in Physical Activity and Sedentary Time

Two studies reported these outcomes [27,28]; however, as different tools were used to measure them, a meta-analysis could not be performed, and the certainty of evidence was considered very low. Bijlholt et al [27] (n=649) reported that the MD in physical activity (metabolic equivalent of task—min/wk) between the groups at 6 and 12 months post partum was not statistically significant (at 6 months: MD 0.052, 95% CI −0.099 to 0.203; *P*=.40; at 12 months: MD 0.144, 95% CI −0.025 to 0.313; *P*=.11). However, in the overweight group, at the 12-month end point, there was a trend toward a change in physical activity, but these results were not statistically significant (MD 0.265, 95% CI −0.001 to 0.531; *P*=.053) was approaching statistical significance. No significant difference was found between the control and intervention groups in sedentary time (min/d) at 6 months (MD −14, 95% CI −39 to 12; *P*=.21) or at 12 months (MD −17, 95% CI −46 to 13; *P*=.22) [27]. Hanafiah et al [28] (n=305) measured physical activity outcomes using the International Physical Activity Questionnaire and found no significant MD (min/wk; MD 0.31; *P*=.13) between the intervention and control groups in sitting (sedentary time).

### Secondary Outcomes

A summary of secondary outcomes can be found in Multimedia Appendix 7 [28,29,31-33].

#### Self-efficacy

Lim et al [31] assessed self-efficacy in regulating exercise using a mobile phone app in postpartum women with recent GDM. The authors reported that at 4 months, women in the intervention group using the *nBuddy* app had higher scores in 2 questions gauging their confidence in being able to perform exercise regularly. These 2 questions addressed how confident participants felt about performing exercise ≥3 a week despite physical discomfort (question 6) and when they had other time commitments (question 11). The intervention group reported higher scores on question 6 (MD 7.94, 95% CI 7.94-1.06, *P*=.02 [unadjusted]) and question 11 (MD 6.64, 95% CI 0.18-13.09; *P*=.04) when compared with the control group. The certainty of evidence was considered to be very low for this outcome.

#### Psychological Outcomes

A total of 3 (43%) studies measured psychological outcomes; however, all used different tools to measure these outcomes. The certainty of evidence was considered to be very low for this outcome. Hanafiah et al [28] assessed changes in stress levels using the Depression Anxiety and Stress Scale–21 Items questionnaire and found no significant differences between the intervention and control groups. Lim et al [31] used the *RAND*-12 Item Health Survey questionnaire to measure the quality of life and found higher emotional distress scores in the intervention group that used the *nBuddy* app (0.21, 95% CI 0.05-0.38). Lim et al [31] hypothesized that emotional distress was related to physical fitness rather than to emotional problems.

Postpartum depression was measured in a study by Jannati et al [30] using the Edinburgh Postnatal Depression Scale. The results showed that the intervention group that used the cognitive behavioral therapy mobile phone app *Happy Mom* had a lower mean Edinburgh Postnatal Depression Scale score (8.18, SD 1.5) than the control group (15.05, SD 2.9), and this was statistically significant (*P=*.001) [30].

#### Evaluation of Intervention: Intervention Compliance, Adherence, and Engagement

Two studies assessed compliance with the *Smarter Pregnancy* intervention, the studies by Oostingh et al [32] and van Dijk et al [33], at 24 weeks. Both studies showed less compliance in the intervention groups (68.5% and 78.9%) than in the control groups (80.8% and 83.5%). Gilmore et al [29] found that postpartum women with high intervention adherence had a reduction in body weight (mean −3.6, SD 1.6 vs mean 1.8, SD 0.9 kg; *P*=.005) and body fat (mean −2.5%, SD 1.0% vs mean 1.7%, SD 0.6%; *P*=.001) when compared with women who received usual care. Lim et al [31] measured user engagement and found that the overall use rate (4-month average) was 65.5%, which the authors claim is significantly higher than other delivery modes, such as face-to-face and telephone-based interventions. Overall, the certainty of evidence was considered to be very low for the outcomes of intervention compliance, adherence, and engagement.

## Discussion

### Summary of the Principal Findings

We aimed to provide a review of the evidence of the effectiveness of mobile phone apps in supporting positive behavior changes in women of reproductive age in the preconception and interconception periods. Despite broadly searching, we identified just 7 studies of low quality. Given the expanding use of and interest in apps as an intervention, we expected to find more studies in this area. We found a wide variation in participant characteristics and outcome measures. The studies assessed anthropometry (clinical), biochemical, self-efficacy, and psychosocial measures in an attempt to determine whether behavior changes had occurred after the intervention. Outcomes measured in the studies included weight control, reduction in adiposity, optimizing health and chronic health diseases, change in risky behaviors (smoking and alcohol use), change in physical activity and sedentary time, self-efficacy, psychological outcomes, and evaluation of adherence, compliance, and engagement with the intervention. We did not find any studies that reported on unintended pregnancy, maternal or neonatal outcomes. All studies compared a mobile phone intervention with standard care or no specific intervention. The end point of the interventions included were 4 to 6 months, with very little follow-up to assess long-term efficacy.

There was considerable heterogeneity across the studies included in the meta-analysis that measured anthropometric measures, which may be related to clinical (preconception and interpregnancy), methodological (differences in study design), and statistical (variations in intervention effects and results) differences. The 3 (43%) studies that measured improved diet and calorie reduction using a validated tool showed improved behavior in those randomized to the mobile phone app; however, the overall results were not significant. The 2 (29%) studies that measured WC and waist to hip ratio showed an increase in WC or no difference (retrospectively). Overall, the evidence is of very low quality. To gain a better understanding of the impact of mobile phone apps as an intervention for weight management, much larger trials that separate preconception and interpregnancy populations and use the same outcome measures are needed.

Studies that measured clinical or biochemical measures had low heterogeneity in the meta-analysis; *P* values of the chi-square tests in the meta-analysis ranged from >.99 to.43. Findings for total cholesterol and HDL were uncertain. The overall effect (*z* test) for total cholesterol was 0.30 (*P*=.76), and for HDL, it was 0.34 (*P*=.74).

### Agreements and Disagreements With Other Studies or Reviews

We did not identify any other published reviews of mobile phone apps that reported evidence of their effectiveness in women of reproductive age in the preconception or interconception period. However, we identified several reviews that have assessed the evidence related to mobile phone apps and behavior change, which are relevant to our findings.

The most relevant to our work is a systematic review by Daly et al [34]. This review aimed to examine the effects of mobile phone app interventions on influencing maternal health behaviors and improving perinatal health outcomes. The main findings from this review are congruent with our findings, in that the authors found it difficult to assess the effect of mobile phone apps on behavior change or outcomes because of the limited number of studies and heterogeneity of outcome measures. Similar to Daly et al [34], we found no evidence of behavior change theory underpinning the design of the app interventions and limited follow-up to gauge longitudinal benefits.

We identified a narrative literature review that aimed to synthesize the latest evidence on the use of mobile phones for weight management. Although not specifically examining women of reproductive age, the review by Ghelani et al [35] suggests that mobile apps may be useful as low-intensity approaches or as additions to standard weight management strategies; however, they should not be a stand-alone weight management intervention. Similar to Ghelani et al [35], we agree that behavioral components such as self-monitoring and tailored feedback are an essential component of any weight management intervention, and optimizing these through technology would only enhance the effect.

Our statistical findings differ from a recent meta-analysis by Islam et al [36], who found that compared with the control group, the use of a mobile phone app was associated with significant changes in body weight (−1.07 kg, 95% CI −1.92 to −0.21; *P*=.01) and BMI (−0.45 kg/m^2^, 95% CI −0.78 to −0.12; *P*=.008). Our findings also differ from a study by Banerjee et al [37], who assessed calorie counting apps and their effectiveness in lifestyle modification and weight management among young Indian adults; this pre-post comparison study found no significant differences in anthropometry or food consumption. It must be noted that neither study specifically examined the population of women of reproductive age, which may be the reason for the difference in the results.

### Strengths of This Review

We did not limit our search by language or use search filters that would reduce returns. Authors from ongoing studies were contacted and asked for an update regarding study progress and preliminary results. Three independent authors conducted the study identification, eligibility assessment, data extraction, and risk of bias assessments.

### Potential Biases in the Review Process

Our review findings are limited by the small number of studies that met the inclusion criteria. Although a comprehensive search was conducted twice, it is possible that relevant studies were missed. In August 2022, while responding to peer-reviewed comments, results from the *Jom Mama* RCT were published by Hanafiah et al [28] in a peer-reviewed journal. Although the results remain the same as in the original trial and we do not believe that this has impacted the quality of this review, this was a deviation from the process.

### Overall Completeness and Applicability of Evidence

Our study found evidence collected from 3 continents. Of the 7 trials, 3 (43%) were conducted in Asia, 3 (43%) were in Europe, and 1 (14%) was in America. We are confident that our study has explored the right participants, interventions, comparisons, and outcomes published and peer reviewed. The intervention apps used in the studies were for research use; therefore, the findings cannot be generalized to commercial app interventions. Overall, we believe that the evidence was complete at the time of writing. However, owing to the fast-paced nature of technology development and this field of research, this review may only serve as a reference point for the potential of smartphone apps as a behavior change intervention.

### Quality of the Evidence

The quality of the evidence presented in this review was very low. This is predominantly because of the risk of bias among included studies, particularly the blinding of participants, personnel, and outcome assessors, and imprecision of results, that is, because of the differences in the total number of participants across studies, differences in the end points of the intervention, and wide CIs.

### Conclusions

#### Implications for Practice

On the basis of the results of our review and the quality of the evidence, health care providers should be aware that the use of mobile phone apps by women of reproductive age may result in little or no difference in positive behavior change. Our review included studies of women seeking health care before conception or between pregnancies (interconception) and does not support the use of mobile phone apps in practice to improve outcomes.

#### Implications for Policy

Currently, there is little evidence to support policy implementation for the use of mobile phone apps as a stand-alone intervention for supporting positive behavior changes during the preconception and interconception periods. No economic analyses (intervention vs normal care) were conducted on any of the interventions used in the included studies; therefore, commercial scaling up of the apps would not be recommended until this is undertaken.

#### Implications for Research

The present body of evidence on mobile phone apps for promoting positive behavior change in women of reproductive age is of low quality, and larger RCTs are required to improve the quality of the evidence. As none of the studies reported on development or cocreation, it would be difficult to replicate the presented studies. The replication of studies with larger sample sizes would potentially provide more information about the long-term efficacy of mobile app interventions and further information on how technology can support individual care plans, particularly for those with health conditions such as diabetes or hypertensive disease.

The challenges of reversing obesity, diabetes, and other chronic diseases in the year before pregnancy suggest that efforts to improve preconception health should be directed at expanding women’s access to primary care. Further research should address this by recruiting individuals from a general population setting such as urban hospitals or community services. This review exposes a research gap in mobile phone apps and their use by women to seek knowledge that informs positive behavior changes. However, this is of direct relevance to health care providers and is not evaluated in this review. Therefore, a future research question is to determine the effects of a mobile phone app targeted at women on a prespecified behavior directly related to reproductive outcomes, such as alcohol consumption or weight maintenance. To address the issue of different outcome measures used by researchers and enhance comparability and reporting, we support a standard set of preconception and interconception measures be developed and adopted. Having a standardized approach not only would help with measuring outcomes but may also benefit the future design of these interventions.

## Appendix

Multimedia Appendix 1 Database search strategy for the initial search in February 2021 and rerun in February 2022.

Multimedia Appendix 2 Studies excluded and reasoning.

Multimedia Appendix 3 Characteristics of the included studies.

Multimedia Appendix 4 Ongoing trials.

Multimedia Appendix 5 Summary of findings table.

Multimedia Appendix 6 Comparisons.

Multimedia Appendix 7 Secondary outcomes.

## Abbreviations

DRS: dietary risk score
GDM: gestational diabetes mellitus
HbA_1c_: glycated hemoglobin
HCP: health care professional
LRS: lifestyle risk score
MD: mean difference
PRISMA: Preferred Reporting Items for Systematic Reviews and Meta-Analyses
RCT: randomized controlled trial
WC: waist circumference
