# Supplementary material for: Addressing Preconception Behavior Change Through Mobile Phone Apps: Systematic Review and Meta-analysis
Source: J Med Internet Res. 2023 Apr 19;25:e41900. doi: 10.2196/41900 (PMC10157458; doi:10.2196/41900)
Supplement: Multimedia Appendix 1 [file jmir_v25i1e41900_app1.docx]

| \| # \| **Searches – Medline** \| **Results** \| \| --- \| --- \| --- \| \| 1 \| Mobile Applications/ \| 6697 \| \| 2 \| (mobile adj3 app*).tw. \| 8127 \| \| 3 \| exp Computers, Handheld/ \| 8601 \| \| 4 \| ((mobile or cell or smart) adj1 phone*).tw. \| 11757 \| \| 5 \| Exp Cell Phone/ \| 8751 \| \| 6 \| 1 or 2 or 3 or 4 or 5 \| 31204 \| \| 7 \| exp Pregnancy/ \| 904760 \| \| 8 \| Pregnant Women/ \| 8743 \| \| 9 \| Reproductive Health/ \| 3575 \| \| 10 \| Preconception Care/ \| 2373 \| \| 11 \| Prenatal Care/ \| 28412 \| \| 12 \| Family Planning Services/ \| 25003 \| \| 13 \| pregnan*.tw. OR reproducti*.tw. \| 508765 \| \| 14 \| matern*.tw. OR mother*.tw. \| 279075 \| \| 15 \| prenat*.tw. \| 99676 \| \| 16 \| pre-nat.tw. \| 10 \| \| 17 \| perinat*.tw. \| 75669 \| \| 18 \| antenat*.tw. \| 37889 \| \| 19 \| preconcept*.tw. \| 5428 \| \| 20 \| pre-concept*.tw. \| 798 \| \| 21 \| 7 or 8 or 9 or 10 or 11 or 12 or 13 or 14 or 15 or 16 or 17 or 18 or 19 or 20 \| 1183882 \| \| 22 \| 6 and 21 \| 1216 \| \| 23 \| ("clinical trial" or "clinical trial, phase i" or "clinical trial, phase ii" or clinical trial, phase iii or clinical trial, phase iv or controlled clinical trial or "multicenter study" or "randomized controlled trial").pt. or double-blind method/ or clinical trials as topic/ or clinical trials, phase i as topic/ or clinical trials, phase ii as topic/ or clinical trials, phase iii as topic/ or clinical trials, phase iv as topic/ or controlled clinical trials as topic/ or randomized controlled trials as topic/ or early termination of clinical trials as topic/ or multicenter studies as topic/ or ((randomi?ed adj7 trial*) or (controlled adj3 trial*) or (clinical adj2 trial*) or ((single or doubl* or tripl* or treb*) and (blind* or mask*))).ti,ab,kw. or ("4 arm" or "four arm").ti,ab,kw. \| 1669054 \| \| 24 \| 22 and 23 \| 296 \| |
| --- | --- | --- | --- | --- | --- | --- | --- | --- | --- | --- | --- | --- | --- | --- | --- | --- | --- | --- | --- | --- | --- | --- | --- | --- | --- | --- | --- | --- | --- | --- | --- | --- | --- | --- | --- | --- | --- | --- | --- | --- | --- | --- | --- | --- | --- | --- | --- | --- | --- | --- | --- | --- | --- | --- | --- | --- | --- | --- | --- | --- | --- | --- | --- | --- | --- | --- | --- | --- | --- | --- | --- | --- | --- | --- | --- |

| # | **Searches – Embase (Ovid)** | **Results** |
| --- | --- | --- |
| 1 | exp mobile application/ | 13781 |
| 2 | (mobile adj3 app*).tw. | 10716 |
| 3 | personal digital assistant/ | 1536 |
| 4 | ((mobile or cell or smart) adj1 phone*).tw. | 16196 |
| 5 | exp mobile phone/ | 31239 |
| 6 | 1 or 2 or 3 or 4 or 5 | 48461 |
| 7 | exp pregnancy/ | 677728 |
| 8 | pregnant woman/ | 83623 |
| 9 | reproductive health/ | 18192 |
| 10 | prepregnancy care/ | 1904 |
| 11 | prenatal care/ | 40525 |
| 12 | family planning/ | 35467 |
| 13 | pregnan*.tw. OR reproducti*.tw. | 651867 |
| 14 | matern*.tw. OR mother*.tw. | 360040 |
| 15 | prenat*.tw. | 128837 |
| 16 | pre-nat.tw. | 42 |
| 17 | perinat*.tw. | 102908 |
| 18 | antenat*.tw. | 54006 |
| 19 | preconcept*.tw. | 8050 |
| 20 | pre-concept*.tw. | 1538 |
| 21 | 7 or 8 or 9 or 10 or 11 or 12 or 13 or 14 or 15 or 16 or 17 or 18 or 19 or 20 | 1209346 |
| 22 | 6 and 21 | 1961 |
| 23 | (Randomized Controlled Trial or Controlled Clinical Trial or Pragmatic Clinical Trial or Equivalence Trial or Clinical Trial, Phase III).pt. | 0 |
| 24 | Randomized Controlled Trial/ | 635873 |
| 25 | exp Randomized Controlled Trials as Topic/ | 192429 |
| 26 | "Randomized Controlled Trial (topic)"/ | 192429 |
| 27 | Controlled Clinical Trial/ | 465234 |
| 28 | exp Controlled Clinical Trials as Topic/ | 200134 |
| 29 | "Controlled Clinical Trial (topic)"/ | 11248 |
| 30 | Randomization/ | 89338 |
| 31 | Random Allocation/ | 85520 |
| 32 | Double-Blind Method/ | 154717 |
| 33 | Double Blind Procedure/ | 179278 |
| 34 | Double-Blind Studies/ | 137773 |
| 35 | Single-Blind Method/ | 39221 |
| 36 | Single Blind Procedure/ | 41249 |
| 37 | Single-Blind Studies/ | 41249 |
| 38 | Placebos/ | 303795 |
| 39 | Placebo/ | 359968 |
| 40 | Control Groups/ | 110506 |
| 41 | Control Group/ | 110506 |
| 42 | (random* or sham or placebo*).ti,ab,hw,kw. | 2128849 |
| 43 | ((singl* or doubl*) adj (blind* or dumm* or mask*)).ti,ab,hw,kw. | 315093 |
| 44 | ((tripl* or trebl*) adj (blind* or dumm* or mask*)).ti,ab,hw,kw. | 1465 |
| 45 | (control* adj3 (study or studies or trial* or group*)).ti,ab,kw. | 1425821 |
| 46 | (Nonrandom* or non random* or non-random* or quasi-random* or quasirandom*).ti,ab,hw,kw. | 57589 |
| 47 | allocated.ti,ab,hw. | 88816 |
|  |  |  |
| 48 | ((open label or open-label) adj5 (study or studies or trial*)).ti,ab,hw,kw. | 66633 |
| 49 | ((equivalence or superiority or non-inferiority or noninferiority) adj3 (study or studies or trial*)).ti,ab,hw,kw. | 13227 |
| 50 | (pragmatic study or pragmatic studies).ti,ab,hw,kw. | 642 |
| 51 | ((pragmatic or practical) adj3 trial*).ti,ab,hw,kw. | 5889 |
| 52 | ((quasiexperimental or quasi-experimental) adj3 (study or studies or trial*)).ti,ab,hw,kw. | 13482 |
| 53 | (phase adj3 (III or "3") adj3 (study or studies or trial*)).ti,hw,kw. | 96028 |
| 54 | 23 or 24 or 25 or 26 or 27 or 28 or 29 or 30 or 31 or 32 or 33 or 34 or 35 or 36 or 37 or 38 or 39 or 40 or 41 or 42 or 43 or 44 or 45 or 46 or 47 or 48 or 49 or 50 or 51 or 52 or 53 | 3169887 |
| 55 | 6 and 21 and 54 | 623 |

| # | **Searches – Web of science** | **Results** |
| --- | --- | --- |
| 1 | TI=((mobile NEAR/3 app*) OR ((mobile or cell or smart)   NEAR/1 phone*)) | [27087](http://apps.webofknowledge.com.ezproxy.lib.uts.edu.au/summary.do?product=WOS&doc=1&qid=2&SID=C3foocZOfiaI3IeSLNm&search_mode=AdvancedSearch&update_back2search_link_param=yes) |
| 2 | AB=((mobile NEAR/3 app*) OR ((mobile or cell or smart)  NEAR/1 phone*)) | [83339](http://apps.webofknowledge.com.ezproxy.lib.uts.edu.au/summary.do?product=WOS&doc=1&qid=3&SID=C3foocZOfiaI3IeSLNm&search_mode=AdvancedSearch&update_back2search_link_param=yes) |
| 3 | #2 OR #1 | [93103](http://apps.webofknowledge.com.ezproxy.lib.uts.edu.au/summary.do?product=WOS&doc=1&qid=4&SID=C3foocZOfiaI3IeSLNm&search_mode=CombineSearches&update_back2search_link_param=yes) |
| 4 | TI=(pregnan* OR matern* OR prenat* OR pre-nat* OR perinat* OR antenat* OR preconcept*  OR pre-concept* OR mother OR reproducti*) | [610349](http://apps.webofknowledge.com.ezproxy.lib.uts.edu.au/summary.do?product=WOS&doc=1&qid=5&SID=C3foocZOfiaI3IeSLNm&search_mode=AdvancedSearch&update_back2search_link_param=yes) |
| 5 | AB=(pregnan* OR matern* OR prenat* OR pre-nat* OR perinat* OR antenat* OR preconcept* OR pre-concept* OR mother OR reproducti*) | [901348](http://apps.webofknowledge.com.ezproxy.lib.uts.edu.au/summary.do?product=WOS&doc=1&qid=6&SID=C3foocZOfiaI3IeSLNm&search_mode=AdvancedSearch&update_back2search_link_param=yes) |
| 6 | #5 OR #4 | [1219,176](http://apps.webofknowledge.com.ezproxy.lib.uts.edu.au/summary.do?product=WOS&doc=1&qid=7&SID=C3foocZOfiaI3IeSLNm&search_mode=CombineSearches&update_back2search_link_param=yes) |
| 7 | #6 AND #3 | [1434](http://apps.webofknowledge.com.ezproxy.lib.uts.edu.au/summary.do?product=WOS&doc=1&qid=8&SID=C3foocZOfiaI3IeSLNm&search_mode=CombineSearches&update_back2search_link_param=yes) |
| 8 | TS=(randomised OR randomized OR randomisation OR randomisation OR placebo* OR (random* AND (allocat* OR assign*) ) OR (blind* AND (single OR double OR treble OR triple) )) | [1222789](http://apps.webofknowledge.com.ezproxy.lib.uts.edu.au/summary.do?product=WOS&doc=1&qid=9&SID=C3foocZOfiaI3IeSLNm&search_mode=AdvancedSearch&update_back2search_link_param=yes) |
| 9 | #8 AND #7 | [259](http://apps.webofknowledge.com.ezproxy.lib.uts.edu.au/summary.do?product=WOS&doc=1&qid=10&SID=C3foocZOfiaI3IeSLNm&search_mode=CombineSearches&update_back2search_link_param=yes) |

| # | **Searches – CINAHL (Ebsco)** | **Results** |
| --- | --- | --- |
| 1 | (MH "Mobile Applications") | 8,131 |
| 2 | (mobile N3 app*) | 10,843 |
| 3 | (MH "Computers, Hand-Held+") | 7,291 |
| 4 | ((mobile or cell or smart) N1 phone*) | 6,431 |
| 5 | (MH "Cellular Phone+") | 7,554 |
| 6 | S1 OR S2 OR S3 OR S4 OR S5 | 24,535 |
| 7 | (MH "Pregnancy") | 202,985 |
| 8 | (MH "Reproductive Health") | 7,481 |
| 9 | (MH "Prepregnancy Care") | 2,007 |
| 10 | (MH "Prenatal Care") | 17,082 |
| 11 | (MH "Family Planning") | 6,499 |
| 12 | pregnan* OR reproducti* | 274,968 |
| 13 | matern* OR mother* | 177,866 |
| 14 | prenat* | 55,233 |
| 15 | pre-nat* | 769 |
| 16 | perinat* | 34,603 |
| 17 | antenat* | 15,127 |
| 18 | preconcept* | 2,770 |
| 19 | pre-concept* | 363 |
| 20 | S7 OR S8 OR S9 OR S10 OR S11 OR S12 OR S13 OR S14 OR S15 OR S16 OR S17 OR S18 OR S19 | 386,401 |
| 21 | S6 AND S20 | 1,207 |
| 22 | TX allocat* random* OR (MH "Quantitative Studies") OR (MH "Placebos") OR TX placebo* OR TX random* allocat* OR (MH "Random Assignment") OR TX randomi* control* trial* OR TX ( (singl* n1 blind*) OR (singl* n1 mask*) ) OR TX ( (doubl* n1 blind*) OR (doubl* n1 mask*) ) OR TX ( (tripl* n1 blind*) OR (tripl* n1 mask*) ) OR TX ( (trebl* n1 blind*) OR (trebl* n1 mask*) ) OR TX clinic* n1 trial* OR PT Clinical trial OR (MH "Clinical Trials+") | 1,512,262 |
| 23 | S21 AND S22 | 412 |

| # | **Searches – Cochrane library**  **Results – 268 total (9 Reviews, 259 Trials)** |
| --- | --- |
| 1 | MeSH descriptor: [Mobile Applications] explode all trees |
| 2 | (mobile NEAR/3 app*):ti,ab |
| 3 | MeSH descriptor: [Computers, Handheld] explode all trees |
| 4 | ((mobile or cell or smart) NEAR/1 phone*):ti,ab |
| 5 | MeSH descriptor: [Cell Phone] explode all trees |
| 6 | #1 OR #2 OR #3 OR #4 OR #5 |
| 7 | MeSH descriptor: [Pregnancy] explode all trees |
| 8 | MeSH descriptor: [Pregnant Women] explode all trees |
| 9 | MeSH descriptor: [Reproductive Health] explode all trees |
| 10 | MeSH descriptor: [Preconception Care] explode all trees |
| 11 | MeSH descriptor: [Prenatal Care] explode all trees |
| 12 | MeSH descriptor: [Family Planning Services] explode all trees |
| 13 | (pregnan* OR reproducti*):ti,ab |
| 14 | (materni* OR mother*):ti,ab |
| 15 | (prenat*):ti,ab |
| 16 | (pre-nat*):ti,ab |
| 17 | (perinat*):ti,ab |
| 18 | (antenat*):ti,ab |
| 19 | (preconcept*):ti,ab |
| 20 | (pre-concept*):ti,ab |
| 21 | #7 OR #8 OR #9 OR #10 OR #11 OR #12 OR #13 OR #14 OR #15 OR #16 OR #17 OR #18 OR #19 OR #20 |
| 22 | #6 AND #21 |
| # | **Searches –** **WHO Global Health Library (renamed to Global Index Medicus)**  **Results – 95** (**No adjacency searching in this database so terms expanded manually)** |
| 1 | (tw:("mobile app*" OR "mobile phone*" OR "smart phone*" OR "cell phone*")) AND (tw:(pregnan* OR reproducti* OR materni* OR mother* OR prenat* OR pre-nat* OR perinat* OR antenat* OR preconcept* OR pre-concept*)) |
