# Supplementary material for: Addressing Preconception Behavior Change Through Mobile Phone Apps: Systematic Review and Meta-analysis
Source: J Med Internet Res. 2023 Apr 19;25:e41900. doi: 10.2196/41900 (PMC10157458; doi:10.2196/41900)
Supplement: Multimedia Appendix 2 [file jmir_v25i1e41900_app2.docx]

| **Study** | **Reason for exclusion** |
| --- | --- |
| Abroms 2017  Abroms 2017a  Actrn 2019  Actrn 2020  Ainscough 2016  Ainscough 2017  Ainscough 2017a  Ainscough 2017b  Ainscough 2020  Altazan 2019  Babalola 2019  Boedt 2019  Boedt 2021  Borgen 2019a  Borgen 2019  Cheng 2016  ChiCtr 2020  ChiCTR2000034263  Christiansen 2019  Coleman Cowger 2018  Crimmins 2019  Ctri 2020  Daly 2018  Darvall 2020  Dehlendorf 2019  deSousaGomes 2019  Dev 2019  Doan 2020  Dodd 2018  Dulli 2019  Earle 2020  Flenady 2019  Franzon 2019  Garnweidner Holme 2020  Garnweidner Holme 2020a  Gilliam 2014  Gilliam 2016  Graham 2014  Greene 2018  Guo 2018  Haidrani 2016  Hantsoo 2018  Herring 2014  Holmes 2020  Ilozumba 2018  Ippoliti 2017  Irct138901212621N 2011  Irct2016101630320N 2016  Irct20170520034052N 2019  Irct20200817048434N 2020  Jacobson 2016  Jiskoot 2017  Kennelly 2016  Kennelly 2017  Kennelly 2017a  Kennelly 2018  Kennelly 2019  Kinser 2021  Krauskopf 2018  Krauskopf 2019  Ledford 2016  Ledford 2018  Lee 2016  Lewkowitz 2020  Lim 2019  Lund 2018  Manlove 2020a  Manlove 2020  MarcanoBelisario 2017  Marin Gomez 2019  Martin 2018  Mascarenhas 2018  Maslowsky 2016  Mauriello 2016  McCarter 2018  McCarthy 2018  McManus 2016  McManus 2017  Mildon 2019  Miremberg 2018  Miremberg 2018a  Moniz 2015  NCT03790449  NCT04242069  NCT03215173  Nct 2015  Nct 2015a  Nct 2016  Nct 2016b  Nct 2016c  Nct 2017  Nct 2017a  Nct 2017b  Nct 2017c  Nct 2018  Nct 2018a  Nct 2018b  Nct 2018c  Nct 2018d  Nct 2019  Nct 2020  Nct 2020a  Nct 2020b  Nicklas 2020  Ntr 2013  Ntr 2016  Nwolise 2016  Olson 2018  Ozcelik 2020  Palmer #2081  Palmer #2082  Patel 2019  Pollak 2014  Pollak 2020  R 2018  Rani 2017  Remick 2013  Ross 2013  Rowe 2019  Sawyer #2044  Seekaew 2015  Sherry 2018  Shorey 2017  Shorey 2018  Shrier 2019  Skar 2018  Skau 2016  Slctr 2017  Smith 2015  Smith 2017  Sridhar 2015  Steinberg 2018  Sukumar 2018  Sun 2019  Sung 2019  Tahata 2017  Takeuchi 2016  Tarqui Mamani 2018  Tebb 2019  Tebb 2019a  Tebb 2020  Teychenne 2021  Tobe 2018  vanderPligt 2018  Vartanian 2020  Wadensten 2019  Wang 2020  Watterson 2015  Wheaton 2018  Wise 2015  Wu 2020  Yew 2020  Yew 2021  Zairina 2016  Zhang 2019  Zulu 2020 | Wrong patient population  Wrong patient population  Wrong intervention  Wrong patient population  Wrong patient population  Wrong patient population  Wrong patient population  Wrong patient population  Wrong patient population  Wrong patient population  Wrong intervention  Protocol  Ongoing trial  Wrong patient population  Wrong patient population  Wrong patient population  Ongoing trial  Ongoing trial  Wrong study design  Wrong intervention  Wrong study design  Wrong patient population  Wrong study design  Wrong patient population  Wrong intervention  Wrong study design  Wrong study design  Wrong patient population  Wrong patient population  Wrong study design  Wrong study design  Wrong patient population  Wrong intervention  Wrong patient population  Wrong patient population  Wrong patient population  Wrong patient population  Wrong study design  Wrong patient population  Wrong patient population  Wrong study design  Wrong patient population  Wrong patient population  Wrong intervention  Wrong study design  Wrong study design  Wrong study design  Wrong patient population  Wrong study design  Wrong patient population  Wrong patient population  Wrong intervention  Wrong patient population  Wrong patient population  Wrong patient population  Wrong patient population  Wrong patient population  Wrong patient population  Wrong patient population  Wrong patient population  Wrong patient population  Wrong patient population  Wrong patient population  Wrong patient population  Protocol  Wrong patient population  Wrong patient population  Wrong patient population  Wrong patient population  Wrong patient population  Wrong patient population  Wrong patient population  Wrong patient population  Wrong patient population  Wrong patient population  Wrong patient population  Wrong intervention  Wrong intervention  Wrong patient population  Wrong patient population  Wrong patient population  Wrong patient population  Ongoing trial  Ongoing trial  Ongoing trial  Wrong patient population  Wrong patient population  Wrong patient population  Wrong patient population  Wrong study design  Ongoing trial  Wrong patient population  Wrong patient population  Wrong intervention  Wrong intervention  Wrong patient population  Wrong intervention  Ongoing trial  Wrong patient population  Wrong intervention  Ongoing trial  Wrong patient population  Wrong study design  Ongoing trial  Wrong patient population  Wrong patient population  Wrong study design  Wrong patient population  Wrong patient population  Wrong study design  Wrong study design  Wrong patient population  Wrong patient population  Wrong patient population  Wrong patient population  Wrong study design  Wrong study design  Wrong study design  Wrong study design  Wrong intervention  Protocol  Wrong patient population  Wrong patient population  Wrong patient population  Wrong patient population  Wrong patient population  Protocol  Wrong patient population  Wrong study design  Wrong study design  Wrong patient population  Wrong study design  Wrong patient population  Wrong patient population  Wrong patient population  Wrong patient population  Wrong patient population  Wrong patient population  Wrong patient population  Wrong patient population  Wrong patient population  Wrong patient population  Wrong intervention  Wrong study design  Wrong patient population  Wrong intervention  Wrong patient population  Wrong study design  Wrong study design  Wrong study design  Wrong intervention  Wrong patient population  Wrong patient population  Wrong patient population  Wrong patient population  Wrong study design |
