# Supplementary material for: Addressing Preconception Behavior Change Through Mobile Phone Apps: Systematic Review and Meta-analysis
Source: J Med Internet Res. 2023 Apr 19;25:e41900. doi: 10.2196/41900 (PMC10157458; doi:10.2196/41900)
Supplement: Multimedia Appendix 3 [file jmir_v25i1e41900_app3.docx]

**Characteristics of studies**

Characteristics of included studies (ordered by study ID**)**

| **Bijlholt 2021 [27]** |  |
| --- | --- |
| ***Study characteristics*** |  |
| Methods | **Study design:** Randomized controlled trial  **Study grouping:** Parallel group |
| Participants | **Baseline Characteristics**  **Participants:** Women with excessive gestational weight gain in the preceding pregnancy (n=1450).  **Age range:** Participants were on average 31.3 years old.  **Recruitment:** Participants were recruited in six hospitals in the region of Flanders, Belgium between May 2017 and April 2019, research midwives approached potential participants two to three days after delivery in the hospital.  **Country:** Belgium.  **Inclusion criteria:** Women with excessive gestational weight gain as defined by the National Academy of Medicine. Women aged 18 or older with a sufficient command of the Dutch language were eligible.  **Exclusion criteria:** Unable or unwilling to give informed consent; no access to the internet; requirement for complex medical diets; history of- or planned bariatric surgery; chronic disorders (e.g. diabetes mellitus type 1 or 2, thyroid disease, renal disease); significant psychiatric disorder; previous stillbirth. Women with twin pregnancies in either the pregnancy preceding the intervention or the subsequent pregnancy are excluded from the study.  **Pre-treatment:** Nil. |
| Interventions | **Intervention:** The lifestyle intervention combined a smartphone application with four face-to-face coaching sessions between six weeks and six months postpartum (n=724).  **Control:** Standard care (n=726). |
| Outcomes | **Primary outcomes:** Eating behavior (restrained eating, uncontrolled eating, emotional eating, high emotional eating), energy intake (kcal), physical activity (MET-minutes per week), sedentary time (minutes per day). Only postnatal outcomes were presented.  **Outcome type:** Dichotomous: dichotomized into low and high emotional eating based on the median score, and logistic regression was performed to test differences between the control group and intervention group.  **Outcome type:** Continuous: energy intake (kcal), physical activity (MET-minutes per week), sedentary time (minutes per day).  **Secondary outcomes:** Nil reported. |
| Identification | **Sponsorship source:** Fund for Scientific Research, ‘Fonds Wetenchappelijk Onderzoek’ (FWO): TBM (Applied Biomedical Research with Primary Social finality) project. Project number FWO: T005116N.  **Country:** Belgium  **Setting:** Participants were recruited in six hospitals in the region of Flanders, Belgium: University Hospital Leuven, University Hospital Antwerp, Gasthuiszusters Hospitals Antwerp, Jessa Hospital in Hasselt, Hospital Oost-Limburg in Genk, and Sint-Franciscus Hospital in Heusden-Zolder.  **Authors name:** Annick Bogaerts  **Institution:** Department of Development and Regeneration, KU Leuven, Herestraat 49, 3000 Leuven, Belgium and Department of Nursing and Midwifery, CRIC Centre for Research & Innovation in Care, University of Antwerp.  **Email:** Annick.Bogaerts@kuleuven.be  **Address:** Faculty of Medicine and Health Sciences, University of Antwerp, 2610 Antwerp, Belgium. |
| Notes | Nil. |

| ***Risk of bias*** |  |  |
| --- | --- | --- |
| **Bias** | **Authors’ judgement** | **Support for judgement** |
| Random sequence generation (selection bias) | Low risk | **Quote:** "The randomization algorithm available in the electronic case report form (eCRF) Castor was used—block randomization with block sizes 4, 6 and 8. At every block generation moment, one of the three-block sizes was randomly selected. When a record was randomized, the allocation was randomly selected from the current block in use. The randomization was stratiﬁed by the hospital." |
| Allocation concealment (selection bias) | High risk | **Quote:** "Participants were enrolled by research midwives, and randomized was performed by the biostatistician within the 1st week postpartum."  **Judgement comment:** Revealed at 6 weeks postpartum after concealment due to coaching sessions. |
| Blinding of participants and personnel (performance bias) | High risk | **Quote:** "Due to the nature of the intervention, participants and study personnel could not be blinded for randomization." |
| Blinding of outcome assessment (detection bias) | Unclear risk | **Quote:** "All caregivers involved in the perinatal care path were blinded for the allocation of a speciﬁc woman."  **Judgement comment:** Not blinded as providing coaching etc v's usual care. |
| Incomplete outcome data (attrition bias) | Low risk | **Judgement comment:** All outcome data appears complete in tables 1-3. Attrition is accounted for in Figure 1. |
| Selective reporting (reporting bias) | Low risk | **Judgement Comment:** Reporting is as per the protocol and methodology. |
| Other bias | High risk | **Quote:** "Some signiﬁcant differences existed between control and intervention group at baseline. More women conceived their pregnancy spontaneously in the control group (92.4%) compared to the intervention group (88.3 %, p = 0.03). Among women with a normal pre-pregnancy BMI, median gestational weight gain was slightly higher in the control group (19 kg, Q1–Q3 17–21 kg) compared to the intervention group (18.2 kg, Q1– Q3 17–20 kg, p = 0.04) (Table 1)." |

| **Gilmore 2017  [29]** |  |
| --- | --- |
| ***Study characteristics*** |  |
| Methods | **Study design:** Randomized controlled trial  **Study grouping:** Parallel group |
| Participants | **Participants:** Overweight or obese postpartum women (n=40).  **Age range:** Women were 23-33 years of age and the median age for the intervention group, E-Moms was 26 and 27.2 for the control group (WIC Moms usual care).  **Recruitment:** An approved brochure was used to recruit women at the first visit they attended postpartum at the Women, infants and children clinic (WIC) at two clinics in Louisiana, USA.  **Country:** USA  **Inclusion criteria:** Postpartum females who gave birth less than 8 weeks ago, ‡18 years old, overweight or obese (body mass index [BMI] ‡25 and <40 kg/m2), certified for WIC post-partum services, and English speaking.  **Exclusion criteria:** Participants were ineligible if they were enrolled in the Nurse-Family Partnership program, had a multiple gestation pregnancy, had a history of psychiatric conditions or chronic disease that can impact body weight, appetite, or intake, type1 diabetes mellitus, or taking medications or supplements to aid in weight loss.  **Pre-treatment:** The participants were recruited, screened, and randomized before7 weeks 6 days postpartum equally to one of two groups: (1) WIC standard care (WIC Moms) and (2) WIC standard care and personalized weight management via a Smart-Phone (E-Moms).  **Post Hoc analysis by adherence:** Within the E-Moms adherence groups, the high adherence group (n=5) was significantly older than the low adherence group (n=7) (32.3–5.4 vs. 23.0–2.7 years; p=0.03), but neither differed significantly from the WIC Moms group (27.2–6.1 years). All of the individuals who were lost to follow-up were black. |
| Interventions | **Intervention:** multi-component  Women received the standard WIC clinic advice (weight and nutritional) and a personalised smartphone weight management program using the app ‘E-Moms’. The app tracked weight and activity and delivered real-time health information and interventionist feedback. Information on diet, physical activity, and behaviour modification (overcoming barriers to lifestyle change, controlling food and hunger cues, mindful eating). A list of behaviour change goals was given to help put the new information into practice (n=19).  **Control:** Usual care that included standard nutritional and weight management advice for postpartum women attending the WIC clinic (n=16). |
| Outcomes | **Primary outcome:** Postpartum weight loss (change in weight following the 4-month intervention).  Clinic Measurements (height, weight, anthropometrics).  **Outcome type:** Continuous: Body fat percentage, waist circumference, hip circumference, and waist-to-hip ratio.  **Reporting:** Fully reported.  **Secondary outcomes:**  Questionnaires.  **Outcome type:** Dichotomous: Improved diet quality (measured using a screening survey). The association between breastfeeding and weight change was measured using the Breastfeeding Self-Efficacy Short Form questionnaire.  **Reporting:** Partially reported.  **Post hoc analysis:** Intervention adherence. |
| Identification | **Sponsorship source:** This work was supported by the United States Department of Agriculture through UCLA Small Grants Program (UCLA Subaward No. 1920 G QA123; L.M.R.) and support of LAG by T32DK064584. This work was supported, in part (J.H.B.), by 1 U54 GM104940 from the National Institute of General Medical Sciences of the National Institutes of Health, which funds the Louisiana Clinical and Translational Science Center.  **Country:** USA.  **Setting:** Louisiana Department of Health and Hospitals; Louisiana Woman, Infants, and Children Capitol City Family Health Clinic and Martin Luther King Community Center.  **Comments:** SmartLoss is a registered trademark of the Louisiana State University System, with the trademarked approach having been developed by Drs. Martin and Redman whom potentially have a vested interest in this intervention being successful.  **Authors name:** Leanne Redman  **Institution:** Pennington Biomedical Research Center  **Email:** leanne.redman@pbrc.edu  **Address:** Pennington Biomedical Research Center 6400 Perkins Road Baton Rouge, LA 70808 |
| Notes | **Participants:**  Recruited during pregnancy but the objective was aimed at decreasing postpartum weight retention.  **Apps as a behaviour change intervention:** Despite near real-time feedback and increased frequency of communication and access to health information, mHealth interventions have the potential to be more passive than in-person interventions. Participants may more easily disengage by ignoring remote communication attempts (i.e., texts, emails, and phone calls) and decrease adherence to recommendations without the accountability of a face-to-face intervention, especially as the study progresses. |

| ***Risk of bias*** |  |  |
| --- | --- | --- |
| **Bias** | **Authors’ judgement** | **Support for judgement** |
| Random sequence generation (selection bias) | High risk | **Quote:** "The participants were recruited, screened, and randomized before 7 weeks 6 days postpartum equally to one of two groups: (1) WIC standard care (WIC Moms) and (2) WIC standard care and personalized weight management via a Smart- Phone (E-Moms)."  **Judgement comment:** Not reported how the randomisation was generated. |
| Allocation concealment (selection bias) | High risk | **Judgement comment:** No description of measures taken for allocation concealment. |
| Blinding of participants and personnel (performance bias) | High risk | **Quote:** "Assessments were performed by study personnel according to standard operating procedures at one of the two collaborating WIC clinics or at Pennington Biomedical Research Center."  **Judgement comment:** Assessments were carried out by research staff and therefore not blinded. No reporting of blinding to group assignment. |
| Blinding of outcome assessment (detection bias) | High risk | **Quote:** "Assessments were performed by study personnel according to standard operating procedures at one of the two collaborating WIC clinics or at Pennington Biomedical Research Center".  **Judgement comment:** Assessments were carried out by research staff therefore not blinded. |
| Incomplete outcome data (attrition bias) | Low risk | **Quote:** “Five participants (WIC Moms, n = 4 and E-Moms, n = 1) were lost to follow-up resulting in an attrition rate of 12.5%”  **Quote:** (Baseline characteristics) “All of the individuals who were lost to follow-up were black. Inclusion of these individuals in the intent-to-treat analysis resulted in a significant difference in race distribution when the groups were stratified by adherence.”  **Quote:** (Weight change) “In the intent-to-treat analysis, weight change within the low adherence group was not significant  (2.4 – 1.2; p = 0.05).”  **Quote:** (Indicators of body composition change) “With baseline values carried forward in the intent-to-treat analysis, estimates and standard errors changed slightly (WIC Moms: 1.7%– 0.54%; p = 0.004 and E-Moms: 0.06 – 0.54; p = 0.91) and resulted in a significant difference between the WIC Moms and E-Moms groups (1.6%– 0.77%; p = 0.04).”  **Judgement comment:** Post randomised withdrawals are explained. In Figure 1 baseline demographics are provided clearly in Table 1. Bodyweight change data across the six weeks is demonstrated in Figure 2. Body composition appears to be complete data (Figure 3). Breastfeeding practices (assessed by questionnaire) are also reported. |
| Selective reporting (reporting bias) | Low risk | **Judgement comment:** All outcomes were reported although no breakdown of breastfeeding rates across the two groups is reported. |
| Other bias | Unclear risk | **Judgement comment:** A small sample size from only 2 sites within the same geographical area and therefore women could openly talk with investigators/data collectors and therefore disclose their treatment group. |

| **Jannati 2020 [30]** |  |
| --- | --- |
| ***Study characteristics*** |  |
| Methods | **Study design:** Randomized controlled trial  **Study grouping:** Parallel group |
| Participants | **Baseline Characteristics**  **Participants:** Postnatal women over the age of 18 years (n=78).  **Age range:** The mean age of participants was 27.65 years (SD=4.2) in the intervention group and 27.39 years (SD=4.8) in the control group.  **Recruitment:** Online announcements via health care centre websites, text message invites to those women who had their mobile phone numbers saved in the health information system, and printed flyers handed directly to women.  **Country:** Iran  **Inclusion criteria:** Postpartum women who had birthed in the previous six months, had at least weekly access to the internet and a mobile phone, and had sufficient Persian language skills to complete a survey, scoring 13 or higher on the Edinburgh Postnatal Depression Scale (EPDS).  **Exclusion criteria:** Not having a smartphone, taking antidepressants or undergoing psychotherapy.  **Pre-treatment:** Before the intervention, the mean EPDS score of mothers in the intervention and control groups were 17.42 ± 2.8 (range: 13–23) and 17.39 ± 2.2 (rang: 9–21). |
| Interventions | **Intervention:** A mobile application (n=38). The ‘Happy Mom’ mobile app was used to deliver cognitive behaviour therapy (CBT) through eight lessons over eight weeks. Lessons were in a linear progression and participants had to complete assignments associated with the lessons.  **Control:** Usual care (n=37). No access to the mobile phone application intervention. |
| Outcomes | **Primary outcomes:** Edinburgh Postnatal Depression Scale (EPDS) score.  **Outcome type:** Continuous: change from baseline.  **Secondary outcomes:** Nil reported. |
| Identification | **Sponsorship source:** This research was funded by Kerman University of Medical Sciences (grant number: 96000642).  **Country:** Iran  **Setting:** Women who attended care from three health care centers in Kerman, Iran.  **Authors name:** Leila Ahmadianb.  **Institution:** Medical Informatics Research Center, Institute for Futures Studies in Health, Kerman University of Medical Sciences, Haft-bagh Highway, PO Box: 7616913555, Kerman, Iran.  **Email:** ahmadianle@yahoo.com, lahmadian@kmu.ac.ir  **Address:** Medical Informatics Research Center, Institute for Futures Studies in Health, Kerman University of Medical Sciences, Haft-bagh Highway, PO Box 7616913555, Kerman, Iran. |
| Notes | **Apps as a behaviour change intervention:** App-based CBT could decrease the EPDS score of all mothers in the intervention group compared to the control group. In other words, the results showed a significant decrease in EPDS score between baseline and 2 months after baseline in the intervention group. On the other hand, there were no significant differences between baseline and 2 months after baseline in terms of EPDS score in the control group. |

| ***Risk of bias*** |  |  |
| --- | --- | --- |
| **Bias** | **Authors’ judgement** | **Support for judgement** |
| Random sequence generation (selection bias) | Low risk | **Quote:** "We did not use blocking or stratiﬁcation during the randomization procedure. Randomization was administered by the medical informatics research center and used a computer-generated code."  **Quote:** "Participants were randomized 1:1 to have immediate mobile application access (intervention group) or no mobile application access (control group)." |
| Allocation concealment (selection bias) | High risk | **Quote:** "We used simple randomisation to allocate mothers to either intervention or control (without any restrictions placed on the sequence)".  **Judgement comment:** “simple” suggests that the allocation was not concealed. |
| Blinding of participants and personnel (performance bias) | High risk | **Quote:** "Because of the nature of the intervention, it was not possible to blind participants regarding their allocation to the intervention or control group."  **Judgement comment:** Non-blinded. |
| Blinding of outcome assessment (detection bias) | High risk | **Quote:** "Because of the nature of the intervention, it was not possible to blind participants regarding their allocation to the intervention or control group."  **Judgement comment:** Non-blinded. |
| Incomplete outcome data (attrition bias)  All outcomes | Unclear risk | **Judgement comment:** Post randomised withdrawals are explained. In Figure 2, three participants did not complete the EPDS (one participant from the intervention group and two from the control group). Intent-to-treat analysis not reported. |
| Selective reporting (reporting bias) | Low risk | **Judgement comment:** All outcome data appears to be reported (Tables 1, 2 & 3). |
| Other bias | High risk | **Protection against contamination**  **Judgement comment:** Women who are depressed are less likely to be involved in studies and complete the lessons in the app intervention and the questionnaires. Women were incentivised to participate. As it was not blinded it is possible that women could openly talk with investigators/data collectors and therefore disclose their treatment group. |

| **Lim 2021 [31]** |  |
| --- | --- |
| ***Study characteristics*** |  |
| Methods | **Study design:** Randomized controlled trial  **Study grouping:** Parallel group |
| Participants | **Baseline Characteristics**  **Participants:** Postnatal women aged ≥21 years who met the eligibility criteria and had been diagnosed with gestational diabetes mellitus (GDM) (n=200).  **Age range:** The mean age of participants was 32.6 years (SD=4.5) in the intervention group and 32.4 years (SD=4.2) in the control group.  **Recruitment:** Electronic medical records of all women in the postnatal ward were screened for eligibility. If eligible they were approached by the study team and consent was obtained.  **Country:** Singapore.  **Inclusion criteria:** Eligible women included postpartum women aged ≥21 years diagnosed with GDM between 24-34 weeks gestation. Women were required to own a smartphone and are able to use an app independently. The first-trimester weight must have been documented at or before 13 weeks.  **Exclusion criteria:** Women with pre-existing type 1 diabetes mellitus orT2DM and women who delivered before 36 weeks.  **Pre-treatment:** Nil  **Post Hoc analysis by adherence:** Nil |
| Interventions | **Intervention:** Mobile application (n=101). A mobile phone application called ‘nBuddy’ (nutritionist buddy). The intervention allowed participants to log their weight, meals and activity. It encouraged web-based interactions with a health team. The app facilitated goal setting and video clips that encouraged diet, exercise and emotional health behaviours.  **Control:** Standard care (N=99). This included a follow-up appointment at 6 weeks postpartum with a clinician for a routine postnatal check. The check included dietary advice and a repeat oral glucose tolerance test (OGTT). |
| Outcomes | **Primary outcome:** The primary outcome was the percentage of women who were able to achieve their first-trimester weight at 4 months postpartum if their first trimester BMI was ≤23 kg/m^2^ or weight loss of at least 5% with respect to first-trimester weight if their first trimester BMI was ≥23 kg/m^2^.  **Outcome type:** Continuous: Clinic Measurements (height, weight, anthropometrics)  **Secondary outcomes:** Several were included: a 75 g 2-hr OGTT, glycated haemoglobin (HbA1c), C-peptide, homeostasis model assessment of insulin resistance, lipid profiles, liver function, high-sensitivity C-reactive protein, and interleukin-6, mean absolute weight loss, breastfeeding status, blood pressure, right-hand grip strength and waist circumference, health-directed behavior scores (heiQ), self-efficacy and RAND-12 questionnaire, caloric and macronutrient intake assessed by a 3-day food.  **Outcome type:** Dichotomous: questionnaires.  **Outcome type:** Continuous: return to first-trimester weight by 4 months.  **Outcome type:** Continuous: 75 g OGTT.  **Outcome type:** Dichotomous: breastfeeding status |
| Identification | **Sponsorship source:** Funding for this study was obtained from a Health Services Research Grant from the National Medical Research Council, Ministry of Health, Singapore.  **Country:** Singapore.  **Setting:** National University Hospital (NUH), Singapore.  **Authors name:** Eu Leong Yong.  **Institution:** Department of Obstetrics and Gynecology, National University Hospital Yong Loo Lin School of Medicine National University of Singapore.  **Email:** obgyel@nus.edu.sg  **Address:** Department of Obstetrics and Gynecology, National University Hospital Yong Loo Lin School of Medicine National University of Singapore5 Lower Kent Ridge Rd Singapore, 119074. |
| Notes | **Apps as a behaviour change intervention:** This study reported that across postnatal lifestyle interventions, the engagement level in this app-based study was significantly higher than in studies relying on telephone-based and face-to-face interventions. Utilization of the app remained constantly high throughout the study period, with 60% usage of the intervention at 4 months. |

| ***Risk of bias*** |  |  |
| --- | --- | --- |
| **Bias** | **Authors’ judgement** | **Support for judgement** |
| Random sequence generation (selection bias) | Low risk | **Quote:** “Participants were randomised at the recruitment visit to the intervention or control arm using a random permuted block design with a block of 4”. |
| Allocation concealment (selection bias) | Low risk | **Quote:** “An independent researcher generated the set of sequences and assigned participants to the intervention or control groups using sequentially numbered sealed opaque envelopes to ensure allocation concealment until the intervention was assigned”. |
| Blinding of participants and personnel (performance bias) | High risk | **Quote:** "Owing to the nature of the intervention, blinding of participants and assessors was not possible”.  **Judgement comment:** Non-blinded. |
| Blinding of outcome assessment (detection bias) | High risk | **Quote:** "Owing to the nature of the intervention, blinding of participants and assessors was not possible”.  **Judgement comment:** Non-blinded. |
| Incomplete outcome data (attrition bias) | Low risk | **Judgement comment:** Post randomised withdrawals are explained. In Figure 1, 11 women (5 intervention and 6 control) were lost to follow-up at week 6 and a further 7 (1 intervention and 6 control) were lost to follow-up at month 4. An intention-to-treat analysis was reported. |
| Selective reporting (reporting bias) | Low risk | **Judgement comment:** The primary outcome was reported (mean anthropometric measurements in the intervention and control group and their mean differences at 4 months postpartum). |
| Other bias | Unclear risk | **Protection against contamination**  **Judgement comment:** The ‘Hawthorne effect’ may have occurred i.e. women in the control arm might have been more likely to modify their health behaviours in response to their awareness of being observed, this may have resulted in bias. Also, women who participated are more likely to be motivated when compared to the general population. |

| **Oostingh 2020 [32]** |  |
| --- | --- |
| ***Study characteristics*** |  |
| Methods | **Study design:** Randomized controlled trial  **Study grouping:** Parallel group |
| Participants | **Baseline Characteristics**  **Participants:** Women undergoing IVF treatment with or without ICSI (n=848).  **Age range:** Women in the study had a median age of 33 (interquartile range 30–36) years.  **Recruitment:** Women were recruited from six IVF centers.  **Country:** Netherlands.  **Inclusion criteria:** Eligible women were 18–45 years of age, had sufficient knowledge or understanding of the Dutch language and were to start their IVF/ICSI treatment within the next 3 months. Male partners were also invited to participate.  **Exclusion criteria:** Women were excluded in case of oocyte donation or adherence to a specific diet (e.g., vegan male partners were also invited to participate if they were not on a specific diet).  **Pre-treatment:** Nil.  **Post Hoc analysis by adherence:** Nil. |
| Interventions | **Intervention:** Smarter Pregnancy program (tailored coaching included a maximum of three e-mails or text messages per week) (n=414).  **Control:** The ‘‘light’’ version of Smarter Pregnancy (did not have tailored information) (n=434). |
| Outcomes | **Primary outcome:** Improvement of inadequate nutritional behaviours based on a reduction of dietary risk score (DRS) 24 weeks after starting the Smarter Pregnancy program.  **Outcome type:** Dichotomous: questionnaires/risk scores.  **Secondary and tertiary outcomes:** Improvement of nutritional and lifestyle behaviours 36 weeks after starting the Smarter Pregnancy program according to the DRS and the lifestyle risk score, compliance to complete the 24 weeks of the coaching program and impact of participation as a couple. Weight and positive pregnancy data were also collected.  **Outcome type:** Dichotomous: questionnaires/risk scores.  **Outcome type:** Continuous: anthropometric measurements. |
| Identification | **Sponsorship source:** Supported by the Department of Obstetrics and Gynecology, Erasmus Medical Center, University Medical Center, Rotterdam, a grant awarded by the Netherlands Organization for Health Research and Development (project no.209040003), and the Erasmus Medical Center Medical Research Advisor Committees‘‘ HealthCare Efficiency Research’’ program.  **Country:** The Netherlands.  **Setting:** IVF clinics  **Comments:** J.M.B. reports personal fees from Advisory board Ferring, Advisory board Merck B.V.  **Authors name:** Prof. dr. R.P.M. Steegers-Theunissen  **Institution:** Department of Obstetrics and Gynecology andbDepartment of Biostatistics, Erasmus Medical Center, University MedicalCenter, Rotterdam.  **Email:** r.steegers@erasmusmc.nl  **Address:** Erasmus MC, Room Ee-2271aP.O. Box 2040 3000 CA Rotterdam, The Netherlands. |
| Notes | Nil |

| ***Risk of bias*** |  |  |
| --- | --- | --- |
| **Bias** | **Authors’ judgement** | **Support for judgement** |
| Random sequence generation (selection bias) | Low risk | **Quote:** "Participating women were randomly assigned to the intervention (regular version of Smarter Pregnancy) or control group (light version of Smarter Pregnancy) in a 1:1 ratio by computer and stratified according to the study center from which they had been recruited. Permuted blocking ensured that the number of women and men from the different study centers was balanced between the treatment groups."  **Quote:** "The Smarter Pregnancy coaching program was available only in the Dutch language, thereby excluding non–Dutch speakers, which gives rise to selection bias." |
| Allocation concealment (selection bias) | Low risk | **Judgement comment:** Assigned and permuted by computer so that balance was achieved. Researchers blinded to participants, partners randomized together. |
| Blinding of participants and personnel (performance bias) | Low risk | **Quote:** "Researchers were blinded to the allocation of the participants. When a woman and her partner participated together, they were both randomized into the same group." |
| Blinding of outcome assessment (detection bias) | Low risk | **Judgement comment:** researchers were blinded. |
| Incomplete outcome data (attrition bias) | Low risk | **Judgement comment:** CONSORT flowchart shows attrition. |
| Selective reporting (reporting bias) | Low risk | **Judgement comment:** All outcome data are presented in tables and supplementary files. |
| Other bias | Unclear risk | **Baseline characteristics**  **Judgement comment:** The study population was highly educated, this may have impacted the results i.e. there may have been a larger improvement in behaviour if less educated |

| **Hanafiah 2022 [28]** |  |
| --- | --- |
| ***Study characteristics*** |  |
| Methods | **Study design:** Randomized controlled trial  **Study grouping:** Two arms |
| Participants | **Baseline Characteristics**  **Participants:** Newly registered married or engaged women (young women prior to their first pregnancy). The woman will be recruited with her spouse, who also will be exposed to the intervention (randomised women at baseline visit n=548).  **Age range:** Women were 20-39 years of age and the median age for the intervention group was 29.1 and 27.9 for the control group.  **Recruitment:** Five designated primary health clinics in Seremban, Malaysia. Other sites in Seremban such as the state marriage registration office, temples and churches or other sites (workplaces, malls, gyms, etc.).  **Country:** Malaysia  **Inclusion criteria:** All women planning to get married in the district of Seremban, in the state of Negeri Sembilan, Malaysia were targeted for participation. The inclusion criteria for participation were: 1) female and between 20-39 years of age; 2) nulliparous; 3) not pregnant at the time of signing the informed consent form; 4) owning a smartphone, with either an Android operating system version 4.1 and above or an  iOS operating system 7.0 and above; and 5) having internet access.  **Exclusion criteria:** Female subject undergoing treatment for type 1 or 2 diabetes mellitus, not residing in the district of Seremban.  **Pre-treatment:** Nil  **Post Hoc analysis by adherence:** Nil. |
| Interventions | **Intervention:** The JOM MAMA intervention has two components. The first component is an interaction with a community health promotor (CHP) who will have three face-to-face meetings, three phone calls and communication through a mobile app group chat. Motivational interviewing techniques were used to support and motivate the participants to live healthier lifestyles. The second component is an E-health platform which consists of two elements: (1) a mobile application in the form of a habit formation application; and (2) a web-based interface (N=272).  **Control:** The control arm received standard care, which is no contact with a CHP and no access to the E-health platform. They received one phone call from a research officer towards the end of the intervention period to remind them of their endpoint visit (N=276). |
| Outcomes | **Primary outcome:** A change in the woman’s waist circumference (WC) from baseline to after 33 weeks.  **Outcome type:** Continuous  **Secondary outcomes:** Differences between intervention and control groups in any change from baseline to after 33 weeks or between the groups at the endpoint in the following variables: weight; body mass index (BMI) following the WHO Asian population cut-off of underweight (<18.5 kg/m2), normal (18.5-22.9 kg/m2), overweight (23.0-27.4 kg/m2  ), obese (≥27.5 kg/m2 ); waist-to-height ratio; waist-to-hip ratio; glycated haemoglobin A1c (HbA1c); fasting lipid profile (total cholesterol, low-density lipoprotein cholesterol, high-density lipoprotein cholesterol, and triglycerides); systolic and diastolic blood pressure; diet as measured by a Food Frequency Questionnaire (FFQ) adapted from a locally validated version [13]; physical activity and sedentary behaviour as measured by the International Physical Activity Questionnaire (IPAQ); and mental health as measured by the Depression Anxiety and Stress Scale 21-item (DASS-21) [12].To evaluate the efficacy of the intervention on dietary habits, physical activity, sedentary behaviour and stress level.  **Outcome type:** Continuous |
| Identification | **Sponsorship source:** The study forms part of the Jom Mama project. This is a public-private partnership with the Ministry of Health Malaysia, Novo Nordisk (Denmark), the University of Southampton (UK), the University of Witwatersrand (South Africa), and the Steno Diabetes Center (Denmark) to address diabetes prevention in Malaysia.  **Country:** Malaysia  **Setting:** The trial was conducted at five sites in the district of Seremban, in the state of Negeri Sembilan in Malaysia.  **Authors name:** Ainul NM Hanafiah.  **Institution:** MRC Developmental Pathways for Health Research Unit, Department of Paediatrics, School of Clinical Medicine, Faculty of Health Sciences, University of the Witwatersrand, Johannesburg, South Africa.  **Email:** jens.aagaard-hansen@regionh.dk  **Address:** Jens Aagaard-Hansen, Health Promotion Research Steno Diabetes Center Copenhagen Herlev, Denmark |

| ***Risk of bias*** |  |  |
| --- | --- | --- |
| **Bias** | **Authors’ judgement** | **Support for judgement** |
| Random sequence generation (selection bias) | Low risk | **Quote:** "At the baseline measurement visit, subjects were randomised into the intervention and control arms at a 1:1 allocation ratio. Random allocation sequences were computer-generated with block sizes of six subjects." |
| Allocation concealment (selection bias) | Low risk | **Quote:** "Each clinic was provided with a sufficient number of identification (ID) numbers with randomisation codes. When a subject is enrolled in the trial, the study nurse assigns the lowest available ID number to the subject from the list of ID numbers. The main implementation partner has prepared the randomisation list and these are distributed to the five designated primary health clinics in Seremban”. |
| Blinding of participants and personnel (performance bias) | High risk | **Judgement comment:** non-blinded, community-based |
| Blinding of outcome assessment (detection bias) | Unclear risk | **Judgement comment:** Not blinded to community health nurses who delivered the intervention, however, the authors were blinded to the randomisation codes. |
| Incomplete outcome data (attrition bias) | Unclear risk | **Judgement comment:** Attrition is documented; however, no information is given regarding if the intention to treat analysis was performed. |
| Selective reporting (reporting bias) | Low risk | **Judgement comment:** Data for all outcomes appears complete. |
| Other bias | Unclear risk | **Judgement comment:** Only married women and Malay couples were recruited, therefore selection bias. Unmarried women may have different characteristics that could influence outcomes. |

| **Van Dijk 2020 [33]** |  |
| --- | --- |
| ***Study characteristics*** |  |
| Methods | **Study design:** Randomized controlled trial  **Study grouping:** Single-centre |
| Participants | **Baseline Characteristics**  **Participants:** Women (and men) if they owned a smartphone with internet access, resided in the Netherlands, and were contemplating pregnancy or already pregnant (<13weeks of pregnancy) (n=218 women, n=36 men).  **Age range:** The mean age of women participants was 30.6 years (interquartile range=5.3) in the intervention group and 30.7 years (interquartile range=5.7) in the control group.  **Recruitment:** Women eligible for inclusion were recruited by a health care professional working in one of the study locations (academic hospital, teaching hospital, midwifery practice, children’s day care or child health centre).  **Country:** Netherlands  **Inclusion criteria:** Women aged 18-45 years who owned a smartphone with internet access, resided in the Netherlands and were contemplating pregnancy or already pregnant (<13weeks of pregnancy).  **Exclusion criteria:** Women were excluded if they had insufficient knowledge and understanding of the Dutch language, if they were being treated by a dietician to lose weight in the context of fertility treatment or if they were on a vegan diet.  **Pre-treatment:** Body mass index (BMI) was not an exclusion criterion. |
| Interventions | **Intervention:** Mobile application ‘Smarter pregnancy’ (version with personalised interaction) (n=109 women). The intervention group received tailored coaching based on their answers to the baseline questionnaire regarding vegetable, fruit, and folic acid supplement intake.  **Control:** Mobile application ‘Smarter pregnancy’ (version with limited functionality and no personalised interaction) (n=109 women). |
| Outcomes | **Primary outcome:** Compliance of all participants, defined as the percentage of participants who completed the online screening at 24 weeks, and degree of improvement in nutrition in women 24 weeks after starting the Smarter Pregnancy program, as reflected by a reduction in the dietary risk score (DRS).  **Outcome type**: Continuous: a reduction in the mean DRS.  **Secondary outcomes:** Mean dietary risk score (DRS) over time in all women who completed the follow-up questionnaire at 36 weeks  Percentage of participants who started the Smarter Pregnancy program.  **Outcome type:** Continuous: a reduction in the mean DRS. |
| Identification | **Sponsorship source:** This research was funded by the Department of Obstetrics and Gynecology, Erasmus Medical Center, University Medical Centre, Rotterdam, the Netherlands; a grant from the Netherlands Organisation for Health Research and Development (ZonMW) HealthCare Efficiency Research program; and the Erasmus Medical Center Mrace Health Care Efficiency Research program.  **Country:** Netherlands  **Setting:** Urban area of Rotterdam, the Netherlands, including one academic hospital, four teaching hospitals, four midwifery practices, and several children’s daycare and child health centers.  **Authors name:** Régine P M Steegers-Theunissen  **Institution:** University Medical Center Rotterdam  **Email:** r.steegers@erasmusmc.nl  **Address:** Department of Obstetrics and Gynaecology Erasmus Medical Center University Medical Center Rotterdam PO Box 2040 Rotterdam Netherlands. |
| Notes | **Apps as a behaviour change intervention:** This study also reported that using a personalized intervention on the mobile phone specifically targeted at identifying and improving preconception risk factors can contribute to lowering the lack of knowledge. |

| ***Risk of bias*** |  |  |
| --- | --- | --- |
| **Bias** | **Authors’ judgement** | **Support for judgement** |
| Random sequence generation (selection bias) | Low risk | **Quote:** "A pre-programmed permuted blocking design (two intervention and two control allocations per block) ensured that the number of women from the different locations was balanced between the two treatment groups". |
| Allocation concealment (selection bias) | Low risk | **Quote:** “Allocation into groups was concealed from the researchers.” |
| Blinding of participants and personal (performance bias) | High risk | **Quote:** “There was no blinding of participants, involved health care professionals, or involved researchers”.  Judgement comment: Non-blinded |
| Blinding of outcome assessment (detection bias) | High risk | **Quote:** “There was no blinding of participants, involved health care professionals, or involved researchers”.  **Judgement comment:** Non-blinded |
| Incomplete outcome data (attrition bias) | Unclear risk | Judgement Comment: Post randomised withdrawals are shown in Figure 1, however, the reason for dropouts is not given. Intention-to-treat is not reported. |
| Selective reporting (reporting bias) | Low risk | **Judgement comment:** All outcome data for women appears to be reported (Tables 1, 2 & Figures 2 &3). |
| Other bias | Unclear risk | **Baseline characteristics**  Judgement Comment: No significant differences between the groups. |
