# Supplementary material for: Addressing Preconception Behavior Change Through Mobile Phone Apps: Systematic Review and Meta-analysis
Source: J Med Internet Res. 2023 Apr 19;25:e41900. doi: 10.2196/41900 (PMC10157458; doi:10.2196/41900)
Supplement: Multimedia Appendix 4 [file jmir_v25i1e41900_app4.docx]

**Characteristics of ongoing trials**

| Trial number | NCT03790449 |
| --- | --- |
| Study name | PreLiFe-RCT |
| Methods | A multicentre RCT |
| Participants | Heterosexual couples starting IVF in Belgian fertility clinics. |
| Interventions | **Control:** Participating couples receive  standard IVF medical treatment. The control group receives a mobile application (app) with treatment information detailing medication instructions and planned appointments but not the PreLiFe programme.  **Intervention:** Both partners of couples randomised to the intervention group receive the new PreLiFe programme. The PreLiFe programme includes a mobile application (PreLiFe-app) with treatment information and tailored advice and skills training on diet, physical activity and mindfulness in combination with interaction with a healthcare professional, trained in motivational interviewing. |
| Outcomes | **Primary outcome:** Cumulative ongoing pregnancy rate (COPR) within 12 months as compared with the control group.  **Secondary outcomes:** Biomedical outcomes are: BMI, waist circumference, IVF discontinuation, clinical pregnancy rate and time to pregnancy. The secondary outcomes in which changes are assessed with patient-reported outcome measures: diet, physical activity, emotional distress and quality of life. |
| Starting date | Trial terminated due to Covid-19 pandemic. All Belgian fertility clinics stopped offering IVF as of March 13th 2020. Recruitment ceased however there was follow-up of study participants already recruited. The primary outcome was adjusted to ‘time to ongoing pregnancy’. |
| Contact information | **Contact:** Christophe Matthys  **Email:** Christophe.matthys@uzleuven.be |

| Trial number | ChiCTR2000034263 |
| --- | --- |
| Study name | Behavior therapy through mobile phone in improving reproductive function for polycystic ovary syndrome |
| Methods | A randomized controlled double-blind study |
| Participants | Women of childbearing age (18-40 yrs) with Polycystic ovary syndrome (PCOS), those who have not had any childbirth plans for the past year, those who are overweight/obese or insulin-resistant, possess and use smartphones proficiently, and are willing to accept nutrition-compliant exercise interventions. |
| Interventions | **Control:** Drug therapy combined with lifestyle adjustment and cloud platform follow-up.  **Intervention:** Mobile terminal behavior intervention combined with lifestyle adjustment, drug treatment and cloud platform follow-up. |
| Outcomes | **Primary outcome:** Ovulation rate.  **Secondary outcomes:** Menstrual cycle, Body index (weight, BMI, waist circumference, hip circumference, WHR, blood pressure), sex hormone, Insulin resistance index (HOMA-IR), antral follicle count, Liver and kidney function, blood lipids, body composition, treatment compliance, adverse reactions, blood samples. |
| Starting date | Not started yet however planning to start 2020-2022. |
| Contact information | **Contact:** Zhang Jing  **Email:** jingzhang110914@126.com |
| Notes | **Query:** Ethics not approved for this study however may have ethics for another related study (ethics dated: 2013-08-26). |

| Trial number | NCT04242069 |
| --- | --- |
| Study name | Healthy for my Baby- A Randomized Controlled Trial Assessing a Preconception Clinically Integrated Technological Intervention to Improve the Lifestyle of Overweight Women and Their Partners |
| Methods | Multi-center open label parallel group RCT |
| Participants | Women in the preconception period will be considered for enrolment if they meet the following eligibility criteria. Inclusion  criteria: (1) Age 18 to 40 years old, (2) BMI ≥ 25 kg/m2 , (3) the participant intends to conceive within 12 months of trial enrolment, (4) access to a smartphone. |
| Interventions | **Control:** Participants randomized to the control group will receive standard advice on healthy lifestyle habits as provided by their usual care provider. Usual care in preconception is the same as that of healthy adults and does not specifically involve access to lifestyle interventions.  **Intervention:** Participants randomized to the intervention group will receive the Healthy for my Baby intervention. Women and their partners will be invited to take part in this lifestyle intervention which includes motivational interviews and daily self-monitoring of lifestyle goals through a mobile phone application. |
| Outcomes | **Primary outcome:** The primary objective of this trial is to evaluate the impact of the intervention on the diet quality of women measured with the Canadian Healthy Eating Index at 2, 4, and 6 months of follow-up.  **Secondary outcomes:** The secondary objectives of the preconception period are to evaluate the impact of the intervention on:  – Urinary metabolomic indicators of women’s dietary exposure at 2 months follow-up,  – The diet quality of male partners at 2, 4, and 6 months follow-up,  – The other lifestyle habits of women and their partners at 3 and 6 months (physical activity, sleep quality, anxious and  depressive symptoms, and quality of life),  – The anthropometric measures of women and their partners at 3 and 6 months (weight, waist circumference, and body  fat percentage), and  – The proportion of women and partners with a weight loss of at least 5% body weight at 3 and 6 months |
| Starting date | Study commenced June 2021. Currently recruiting, estimated completion date December 2022 (ClinicalTrials.gov). |
| Contact information | **Contact:** Ana C Colmenares  **Email:** [ensantepourbebe@usherbrooke.ca](mailto:ensantepourbebe@usherbrooke.ca) |

| Trial number | NCT03215173 |
| --- | --- |
| Study name | Fit After Baby: Increasing Postpartum Weight Loss in Women at Increased Risk for Cardiometabolic Disease (FAB) |
| Methods | Parallel group RCT |
| Participants | Women aged 18-45, 4-6 weeks postpartum Body Mass Index (BMI): 26- 45 kg/m2 (≥24 for Asians), positive history of one or more of the following complications in most recent singleton or twin pregnancy: a) Gestational diabetes mellitus, b) Preeclampsia, c) gestational hypertension, d) pre-term delivery (32-37 weeks), e) small for gestational age (<10th percentile for gestational age). Also, need access to and be willing to use wi-fi enabled iPhone. |
| Interventions | **Control:** Text4Baby group will receive text messages from the free Text4Baby program  **Intervention: ‘**Fit After Baby’ group will receive a mobile health lifestyle intervention to increase postpartum weight loss, increase postpartum physical activity, and improve postpartum diet. |
| Outcomes | **Primary outcomes:** 1) Change in weight loss from baseline to one year postpartum  documentation of any change in weight at one year postpartum compared to weight at baseline, 6 weeks, 6 months and 12 months. 2) Change in postpartum weight retention documentation of any change in postpartum weight at one year postpartum compared to pre-pregnancy weight at 6 weeks, 6 months and 12 months.  **Secondary outcomes:**   1. Evaluation of Subject Satisfaction 2. Adherence to self monitoring 3. Use of app 4. Number of interactions with lifestyle coach 5. Change in waist circumference 6. Change in fasting glucose 7. Change in HbA1c 8. Change in fasting insulin 9. Change in adiponectin 10. Change in lipids 11. Change in blood pressure 12. Change in hsCRP 13. Change in postnatal depression score 14. Change in Physical activity 15. Change in Social Support Social 16. Change in Self-Efficacy 17. Change in Perceived 18. Change in dietary 19. Change in breastfeeding status 20. Change in readiness to change score |
| Starting date | Study commenced September 2017 and completed November 2020 ClinicalTrials.gov). Author contacted for further information, manuscript drafted for submission to a scientific journal. |
| Contact information | **Contact:** Jacinda Nicklas  **Email:** [JACINDA.NICKLAS@CUANSCHUTZ.EDU](mailto:JACINDA.NICKLAS@CUANSCHUTZ.EDU) |
