# Supplementary material for: Addressing Preconception Behavior Change Through Mobile Phone Apps: Systematic Review and Meta-analysis
Source: J Med Internet Res. 2023 Apr 19;25:e41900. doi: 10.2196/41900 (PMC10157458; doi:10.2196/41900)
Supplement: Multimedia Appendix 5 [file jmir_v25i1e41900_app5.docx]

| Outcome № of participants (studies) | **Anticipated absolute effects (95% CI)** | | | Certainty |
| --- | --- | --- | --- | --- |
|  | **Without a mobile phone app** | **With mobile phone apps** | **Difference** |  |
| 1. Energy intake  № of participants: 937 (2 RCTs) | The mean energy intake was **1666** kcal | The mean energy intake was **1322** kcal | MD **140.89 kcal fewer** (-190.19 fewer to -91.59 fewer) | ⨁◯◯◯ Very low^b,c,e^ |
| 1. Weight loss  № of participants: 529 (3 RCTs) | The mean weight loss was **0.18** kg | The mean weight loss was **0.77** kg | MD **0.78 kg fewer** (-1.20 fewer to -0.36 fewer) | ⨁◯◯◯ Very low^a,b,c,d,e^ |
| 1. Body fat loss (BMI) № of participants: 340 (2 RCTs) | The mean body fat loss was **0.52** % | The mean body fat loss was **0.12** % | MD **0.32 % lower** (-0.55 lower to -0.09 lower) | ⨁◯◯◯ Very low^a,b,c,e^ |
| 1. Blood pressure - Systolic  № of participants: 529 (3 RCTs) | The mean blood pressure - Systolic was **109.4** mmHg | The mean blood pressure - Systolic was **110.6** mmHg | MD **1.63 mmHg higher** (-0.42 lower to 3.68 higher) | ⨁◯◯◯ Very low^a,c^ |
| 1. Blood pressure - Diastolic  № of participants: 340 (2 RCTs) | The mean blood pressure - Diastolic was **72.4** mmHg | The mean blood pressure - Diastolic was **73.5** mmHg | MD **1.33 mmHg higher** (-0.77 lower to 3.42 higher) | ⨁◯◯◯ Very low^a,c^ |
| 1. HbA1c assessed with: % № of participants: 494 (2 RCTs) | The mean HbA1c was **5.2** % | The mean HbA1c was **5.3** % | **0.1 % higher** (0.04 higher to 0.16 higher) | ⨁◯◯◯ Very low^a,b,c^ |
| 1. Total cholesterol assessed with: mmol/L № of participants: 494 (2 RCTs) | The mean total cholesterol was **5.02** mmol/L | The mean total cholesterol was **5.00** mmol/L | **0.02 mmol/L higher** (-0.13 lower to 0.18 higher) | ⨁◯◯◯ Very low^a,c,d^ |
| 1. HDL assessed with: mmol/L № of participants: 494 (2 RCTs) | The mean HDL was **1.52** mmol/L | The mean HDL was **1.53** mmol/L | **0.01 mmol/L higher** (-0.06 lower to 0.08 higher) | ⨁◯◯◯ Very low^a,c,d^ |

#### Explanations

a. Downgraded for unclear risk of bias

b. P-value <0.05, therefore, null hypothesis rejected

c. Endpoint differences in measure variable

d. 95% CI overlaps no effect

e. Unexplained heterogenity of results
