# Supplementary material for: Addressing Preconception Behavior Change Through Mobile Phone Apps: Systematic Review and Meta-analysis
Source: J Med Internet Res. 2023 Apr 19;25:e41900. doi: 10.2196/41900 (PMC10157458; doi:10.2196/41900)
Supplement: Multimedia Appendix 6 [file jmir_v25i1e41900_app6.docx]

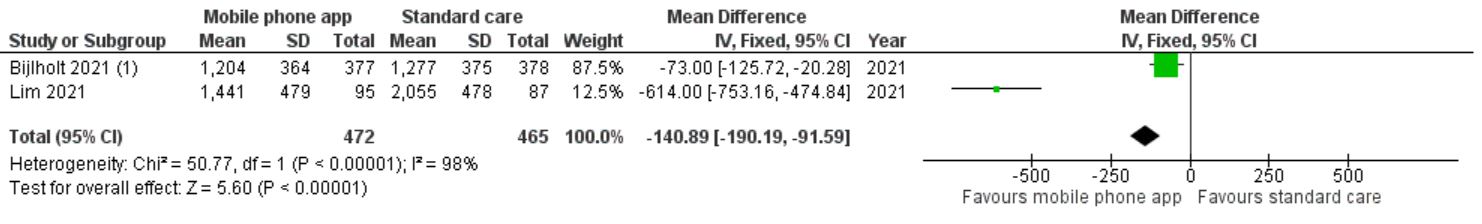


Figure S1. Comparison: mobile phone app versus standard care. Outcome: mean difference in mean calories (kg) 4-8 months from the baseline.


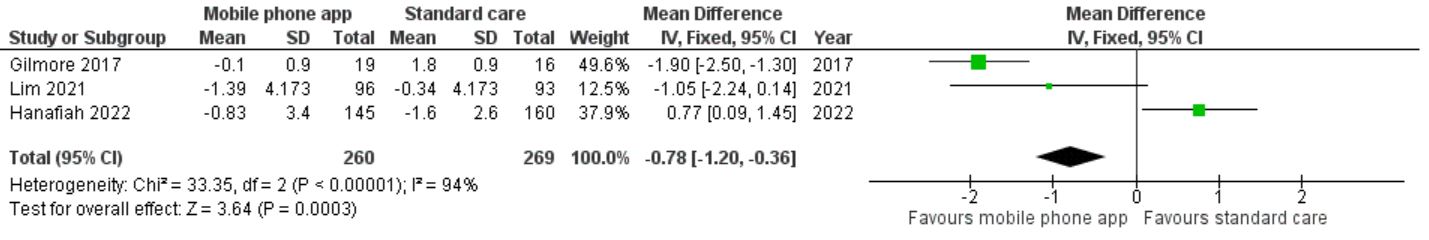


Figure S2. Comparison: mobile phone app versus standard care. Outcome: mean difference in weight loss (kg) 4-8 months from the baseline.


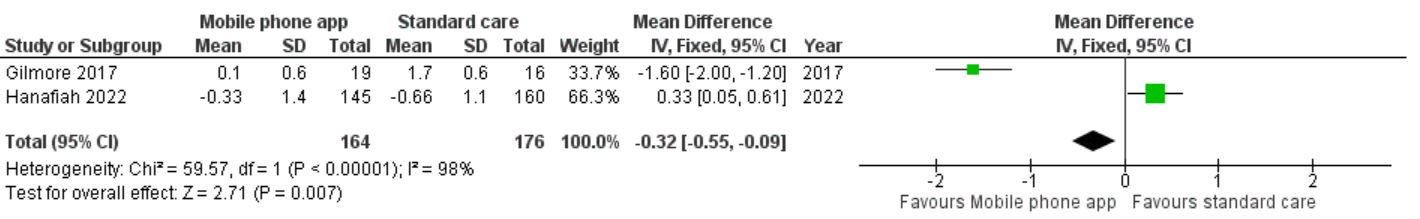


Figure S3. Comparison: mobile phone app versus standard care. Outcome: mean difference in body fat (%) 4-8 months from the baseline.


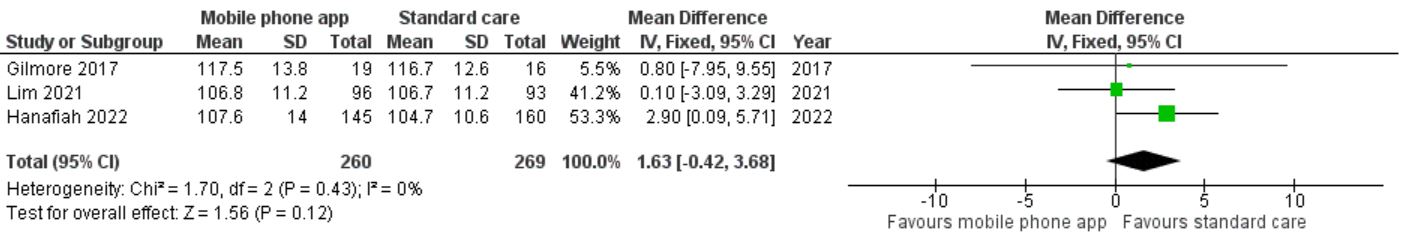


Figure S4. Comparison: mobile phone app versus standard care. Outcome: mean difference in systolic blood pressure (mmHg) 4-8 months from the baseline.


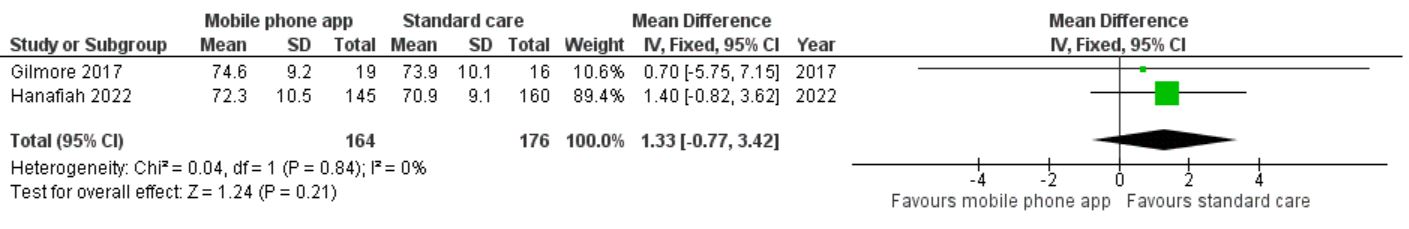


Figure S5. Comparison: mobile phone app versus standard care. Outcome: mean difference in diastolic blood pressure (mmHg) 4-8 months from the baseline.


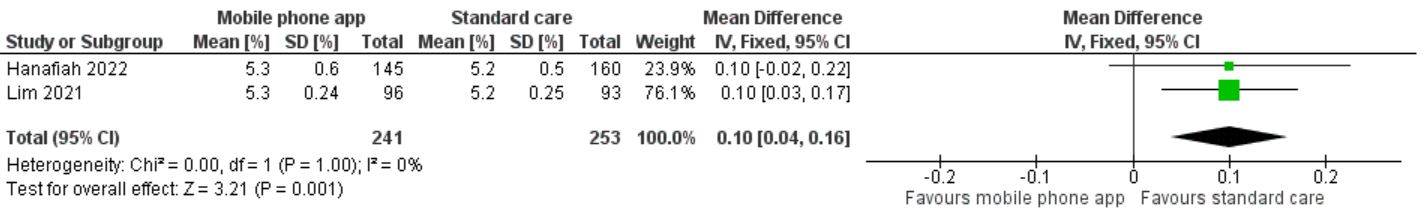


Figure S6. Comparison: mobile phone app versus standard care. Outcome: mean difference in HbA1c (%) 4-8 months from the baseline.


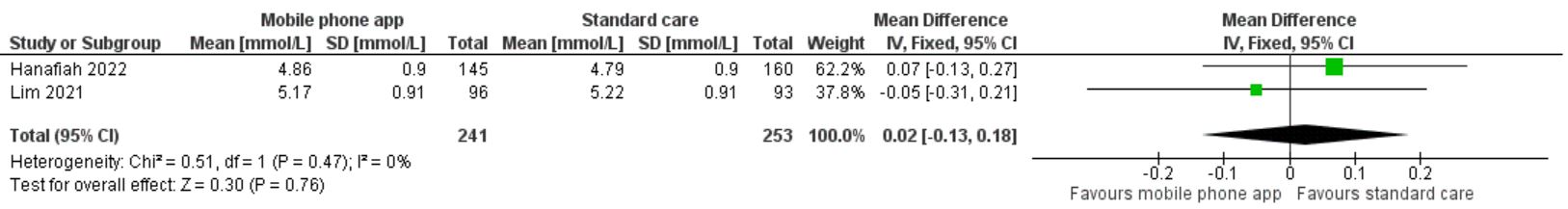


Figure S7. Comparison: mobile phone app versus standard care. Outcome: mean difference in total cholesterol (mmol/L) 4-8 months from the baseline.


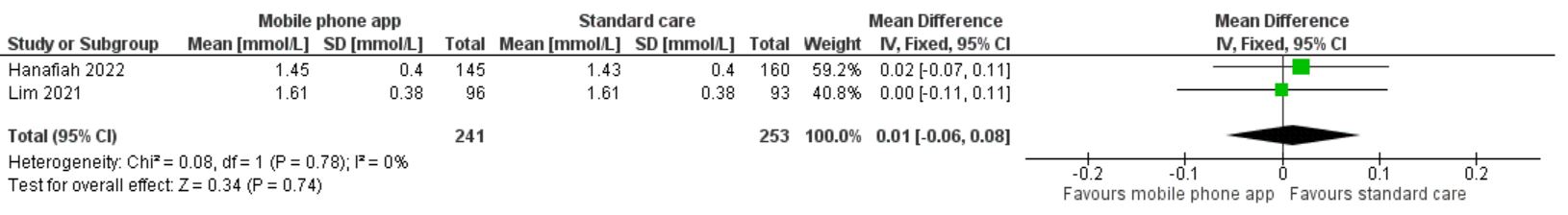


Figure S8. Comparison: mobile phone app versus standard care. Outcome: mean difference in HDL (mmol/L) 4-8 months from the baseline.
