# Supplementary material for: Addressing Preconception Behavior Change Through Mobile Phone Apps: Systematic Review and Meta-analysis
Source: J Med Internet Res. 2023 Apr 19;25:e41900. doi: 10.2196/41900 (PMC10157458; doi:10.2196/41900)
Supplement: Multimedia Appendix 7 [file jmir_v25i1e41900_app7.docx]

| **Outcomes** | N | Intervention | N | Control | P-value (where reported) |
| --- | --- | --- | --- | --- | --- |
| **Gilmore et al. (2017)** | | | | | |
| **Evaluation of intervention (Intervention adherence)** | | | | | |
| High^a^ (weight in Kg) | 5 | -3.6 ± 1.6 | 16 | -5.4 ± 1.8 | 0.03 vs 0.005 |
| High^a^ (body fat in %) | 5 | -2.5 ± 1.0 | 16 | 1.7 ± 0.6 | 0.02 vs 0.001 |
| High^a^ (hip circumference in cm) | 5 | -5.0 ± 1.7 | 16 | NR | 0.006 |
| Medium^b^ (weight in Kg) | 7 | -0.4 ± 1.3 | 16 | NR | 0.75 vs 0.17 |
| Medium^b^ (hip circumference in cm) | 7 | 0.96 ± 1.44 | 16 | NR | 0.51 |
| Low^c^ (weight in Kg) | 7 | 2.7 ± 1.3 | 16 | NR | 0.04 vs 0.56 |
| Low^c^ (hip circumference in cm) | 7 | 2.8 ± 1.4 | 16 | NR | 0.06 |
| **Hanafiah et al. (2022)** | | | | | |
| **Psychosocial outcomes (DASS-21)**^d^ | | | | | |
| Depression: Normal | 145 | 114 (78.6) | 160 | 124 (77.5) | 0.595 |
| Moderate | 145 | 29 (20) | 160 | 31 (19.4) | NR |
| Severe | 145 | 2 (1.4) | 160 | 5 (3.1) | NR |
| Anxiety: Normal | 145 | 77 (53.1) | 160 | 81 (50.6) | 0.635 |
| Moderate | 145 | 50 (34.5) | 160 | 53 (33.1) | NR |
| Severe | 145 | 18 (12.4) | 160 | 26 (16.3) | NR |
| Stress: Normal | 145 | 119 (82.1) | 160 | 124 (77.5) | 0.190 |
| Moderate | 145 | 23(15.9) | 160 | 26 (16.3) | NR |
| Severe | 145 | 3 (2.1) | 160 | 10 (6.3 | NR |
| **Oostingh et al. (2020)** | | | | | |
| **Evaluation of intervention (Compliance)**^d^ | | | | | |
| Program completed (24 weeks) | 308 | 211 (68.5) | 318 | 257 (80.8) | NR |
| **van Dijk et al. (2020)** | | | | | |
| **Evaluation of intervention (Compliance)**^d^ | | | | | |
| Program completed (24 weeks) | 109 | 86 (78.9) | 109 | 91 (83.5) | 0.95 |
| **Lim et al. (2021)** | | | | | |
| **Successful initiation of breastfeeding (Mean difference (95% CI)** | | | | | |
| Exclusive breastfeeding (days): Unadjusted: 0.95 (0.53-1.70) | 96 | 51.6 | 93 | 52.9 | 0.86 |
| Adjusted: 1.26 (0.41-3.86) | 96 | 50.5 | 93 | 48.3 | 0.68 |
| **Evaluation of intervention (User engagement)** | | | | | |
| Overall utilization rate: 4-month average | 101 | 65.5 (29.0) | NR | NR | NR |

Notes: Unless otherwise stated, values reported for intervention and control groups are presented as mean ± standard error. ^a^ High adherence was meeting >70% (5 for more days of engagement per week). ^b^Medium adherence was meeting 40.1-70% (3 days or engagement per week). ^c^Low adherence was meeting ≤40% (1-3 days of engagement per week). ^d^ Reported as frequency and percentage. ^e^Percentage of days an app component was used per user and mean (SD). BP=Blood pressure. DASS-21=Depression Anxiety and Stress Scale 21 items. NR=Not reported
